# Tumor-promoting UBR4 coordinates impaired mitophagy–associated senescence and lung adenocarcinoma pathogenesis

**DOI:** 10.1073/pnas.2425015122

**Published:** 2025-06-18

**Authors:** Dawon Jeong, Seo Hyeong Park, Jiwon Kim, Hyeyoon Kim, Yejin Jang, Jaemoon Koh, Yoon Kyung Jeon, Takafumi Tasaki, Yong Tae Kwon, Dohyun Han, Sung-Yup Cho, Min Jae Lee

**Affiliations:** ^a^Department of Biochemistry and Molecular Biology, Seoul National University College of Medicine, Seoul 03080, Korea; ^b^Department of Biomedical Sciences, Seoul National University Graduate School, Seoul 03080, Korea; ^c^Genomics Core Facility, Transdisciplinary Research & Collaboration Division, Biomedical Research Institute, Seoul National University Hospital, Seoul 03080, Korea; ^d^Proteomics Core Facility, Transdisciplinary Research & Collaboration Division, Biomedical Research Institute, Seoul National University Hospital, Seoul 03080, Korea; ^e^Biological Sciences Division, Pacific Northwest National Laboratory, Richland, WA 99354; ^f^Department of Pathology, Seoul National University College of Medicine, Seoul 03080, Korea; ^g^Department of Life Sciences, Kanazawa Medical University, Ishikawa 920-0293, Japan; ^h^Department of Pathology and Laboratory Medicine, University of California, Los Angeles, CA 90095; ^i^Ischemic/Hypoxic Disease Institute, Convergence Research Center for Dementia, Medical Research Center, Seoul National University, Seoul 03080, Korea

**Keywords:** UBR4, lung adenocarcinoma, oncogene, senescence, mitophagy

## Abstract

Cellular senescence, an irreversible cell cycle arrest, plays a pivotal role in development, aging, and tumor suppression. However, the fundamental pathway coordinating senescence and neoplastic transformation remains unclear. Here, we describe the tumorigenic involvement of ubiquitin protein ligase E3 component n-recognin 4 (UBR4), an E3 ubiquitin ligase of the N-degron pathway, in lung adenocarcinoma (LUAD). Public genome databases revealed high UBR4 expression in LUAD patients, associated with a dysregulated cell cycle and impaired mitochondrial homeostasis. *UBR4* knockout (ΔUBR4) in A549 lung cancer cells induced cellular senescence with defective mitochondria. Restoration of UBR4 or antioxidant treatment reversed the ΔUBR4 phenotypes caused by impaired mitophagy. Mitochondrial stress exacerbated mitochondrial dysfunction in ΔUBR4 cells, contributing to diverse cellular phenotypes. Additionally, ΔUBR4 cells exhibited substantially slow tumor growth in mouse xenograft models. In LUAD patients, UBR4 levels correlated with tumor stage, mitophagy markers, and poor survival. These findings suggest a tumor-promoting function of UBR4 in LUAD by regulating mitochondrial quality control. Further research into the pharmacological inhibition of UBR4 could open promising avenues for developing effective antitumor therapies targeting LUAD.

## Significance

Our study identifies a role of UBR4 in regulating mitochondrial quality, cellular aging, and tumor growth in lung adenocarcinoma (LUAD). UBR4 enables mitophagy to maintain healthy mitochondria and support stress adaptation. Loss of UBR4 causes mitochondrial dysfunction, excessive reactive oxygen species, cell cycle arrest, and senescence while avoiding apoptosis. Interestingly, UBR4 inactivation impairs tumor growth by disrupting mitochondrial dynamics but promotes inflammation linked to senescence. These findings highlight UBR4 as a crucial regulator of cellular fate, balancing apoptosis and senescence during stress. Targeting the UBR4–mitophagy pathway presents promising therapeutic opportunities for LUAD and other diseases associated with mitochondrial dysfunction, potentially paving the way for innovative cancer treatments.

Cellular senescence is a state of terminal cell cycle arrest in which premalignant cells lose their ability to proliferate (1). Unlike quiescent cells, which are in the G0 phase and can resume proliferation in response to mitogenic signals, senescent cells remain arrested in the G1 or G2 phase. Under senescence, cells become multinucleated and enlarged, distinguishing them from normal and quiescent cells (2). Phenotypically, senescence is accompanied by elevated senescence-associated β-galactosidase (SA-β-gal) activity, senescence-associated secretory phenotypes (SASPs), epigenetic reprogramming, autophagy dysfunction, and proinflammation (1, 3). Senescence can be triggered by various internal and external stressors, including chronic DNA damage, telomere shortening, mitochondrial dysfunction, proteotoxicity, and oxidative stress (4, 5). It is widely recognized as a natural barrier against tumorigenesis because of its stalled proliferation, primarily driven by hyperactivation of cyclin-dependent kinase inhibitor 1A (CDKN1A)/p21 and CDKN2A/p16 (6). Many E3 ubiquitin (Ub) ligases, including the anaphase-promoting complex/cyclosome and Skp1–Cul1–F-box complexes, coordinate Ub-dependent proteolysis of cyclins and CDKs, ensuring precise cell cycle progression in eukaryotes (7).

Mitochondrial function is integral to cellular homeostasis, and its perturbation leads to various consequences, including apoptosis and senescence, depending on the cell/tissue type, physiological status, and stressors (4). Senescent cells are often hypermetabolic because of increased oxygen consumption via glycolysis as well as reactive oxygen species (ROS) (8, 9). Conversely, hyperactive mitochondrial respiration causes an imbalance in cellular energy and global oxidative stress, leading to genomic DNA damage, tumor protein p53 (TP53) activation, and cell cycle arrest (10). Therefore, impaired mitochondria can be both causal and consequential factors of senescence (11). Autophagy and senescence, the primary regulators of homeostatic stress responses, are inextricably linked (12, 13). Autophagy deficiency can cause early senescence in a ROS-dependent manner, whereas autophagy activation suppresses senescence phenotypes (14, 15), supporting the notion that autophagy acts as a senescence-protective mechanism. In addition, many studies have reported a decrease in mitochondrial fission associated with senescence (16, 17). The resultant interconnected mitochondria are more resistant to both mitophagic clearance and apoptotic stimuli, such as oxidative stress. Nevertheless, the common denominators (or pathways) that coordinate cell survival and death during stress are yet to be identified.

Ubiquitin protein ligase E3 component N-recognin 4 (UBR4) was initially identified as a member of the E3 Ub ligase family involved in the “N-degron” pathway (18), in which the in vivo half-life of a protein is primarily determined by the identity of its N-terminal residues. UBR proteins (UBR1–UBR7) share a 70-residue two-Zn^2+^-coordinated UBR box that recognizes and ubiquitinates N-degrons (19). The N-degron pathway is also implicated in autophagy-mediated proteolysis, as the autophagic cargo receptor sequestosome 1 (SQSTM1)/p62 binds N-degrons via its UBR-like ZZ domain (20, 21). Unlike other UBR proteins, UBR4 ubiquitinates substrates via an unusual “hemiRING” zinc finger (22), mediating distinct modes of biochemical action. For example, UBR4 generates Lys11/Lys48-branched poly-Ub chains in an N-degron-independent manner (23). The physiological function of UBR4, as well as its direct substrates, remains largely unknown, although UBR4 has been reported to regulate apoptosis, autophagy, membrane morphogenesis, and endolysosomal pathways in *Drosophila* and mice (24–26). More recent studies demonstrated that *UBR4* depletion markedly delays cell cycle in the G1 phase (27). Furthermore, UBR4 is a critical sensor in mitigating various stressors, particularly proteotoxic proteins imported into the mitochondria (28).

We aimed to understand the functional significance of UBR4 in tumorigenesis, particularly in lung adenocarcinoma (LUAD), where *UBR4* messenger RNA (mRNA) expression is significantly enhanced; however, its underlying mechanism remains unknown. LUAD is the most common subtype of non–small cell lung cancer and is characterized by a high incidence of metastasis, recurrence, and a 5-y survival rate of less than 20% (29, 30). We found that *UBR4*–deleted A549 human lung cancer cells underwent senescence potentially through the TP53–CDKN1A axis. *UBR4*-null cells exhibited impaired oxidative phosphorylation (OXPHOS), elevated ROS levels, and dysfunctional mitochondria. Notably, adding back UBR4 into the knockout cells effectively reversed these traits, indicating that UBR4 acts as a direct suppressor of cellular senescence. Using mouse xenograft models, we found that cells lacking UBR4 developed considerably slower than those from wild-type (WT) controls. Consistently, tissue microarray (TMA) analysis of human tumor tissues revealed that UBR4 expression significantly correlated with LUAD progression and with levels of PTEN-induced putative kinase 1 (PINK1), a crucial mediator of mitophagy-mediated mitochondria quality control. These results revealed the pathological mechanisms underlying UBR4-mediated tumorigenesis in LUAD, highlighting the therapeutic potential of targeting UBR4 as a feasible method for lung cancer treatment. (Please note that this study used the official human gene and protein names determined by the HUGO gene nomenclature committee; https://www.genenames.org/).

## Results

### Clinical Evidence Identifies a Significantly Higher *UBR4* Expression in LUAD.

**Fig. 1.**
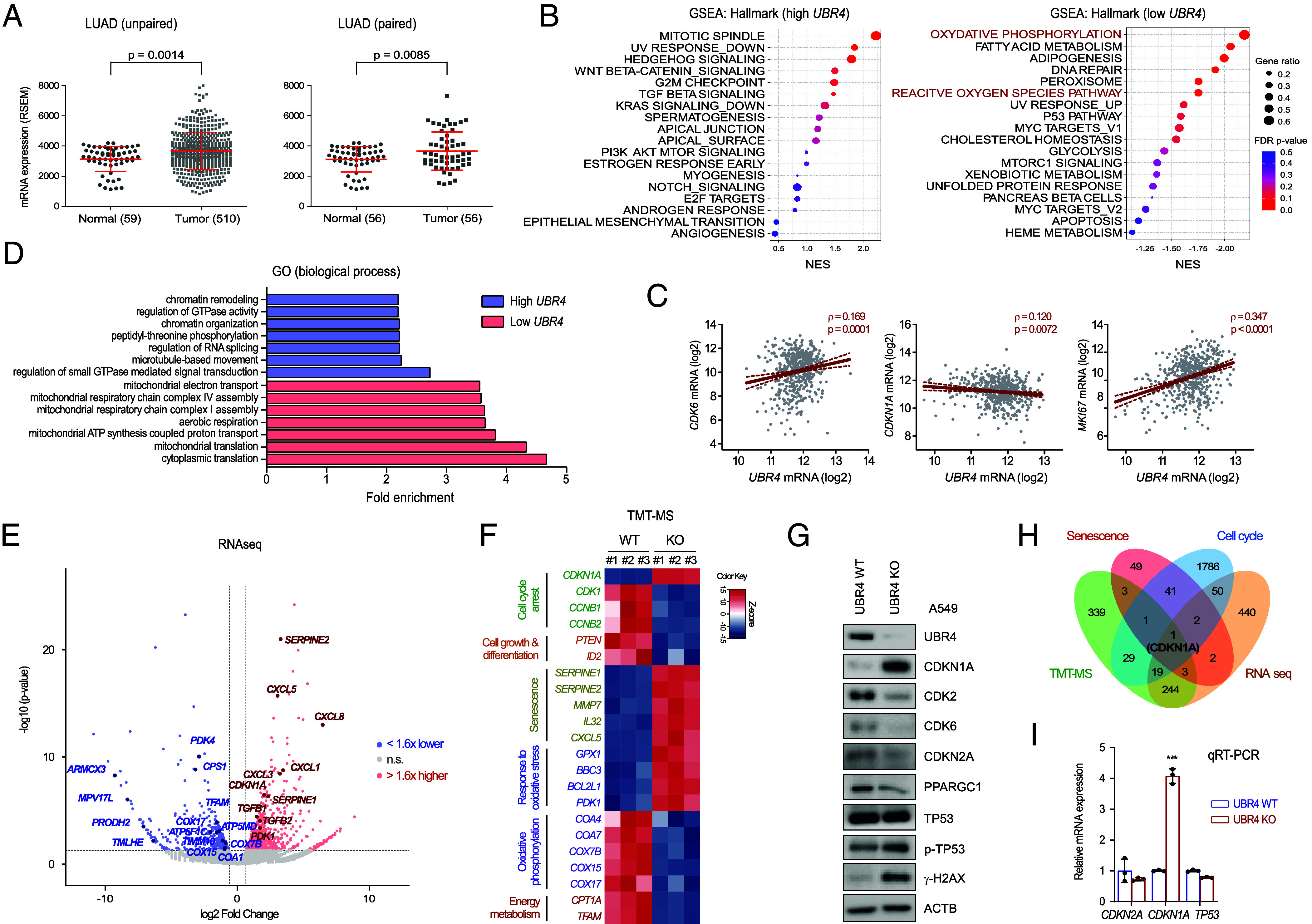
Identification of the pathological link between the *UBR4* expression and LUAD. (*A*) Dot plots with mean and SD bars, comparing *UBR4* mRNA expression levels between normal and tumor tissues in patients with LUAD. Data were obtained from cBioportal (https://www.cbioportal.org/). *Left:* Analysis of all LUAD data from TCGA (normal, n = 59; tumor, n = 510). *Right:* Comparison of paired samples (n = 56) was performed using a two-tailed unpaired or paired Student’s *t* test. (*B*) GSEA showing DEG pathways in LUAD tissues with high (first quartile; *Left*) and low (fourth quartile; *Right*) *UBR4* expression. Top hallmark pathways are displayed with NESs on the x-axis. Dot size indicates the “gene ratio” (k:n), where k is the number of core enrichment genes and n is the total number of genes in each set. Dot colors denote FDR-corrected *P*-values. (*C*) Scatter plot illustrated correlations between *UBR4* expression and cell cycle-related mRNAs (*CDKN1A/p21*, *CDK6*, or *MKI67*) in LUAD patients. Solid lines depict regression lines, whereas dashed lines represent 95% CI (n = 510, ρ- and *P*-values from Spearman’s correlation analysis). (*D*) GO enrichment analysis using the DAVID database, highlighting genes significantly coexpressed with *UBR4*. Bar plots displayed biological process (BP) terms ordered by fold enrichment (x-axis) with blue indicating negative and red indicating positive associations. (*E*) Volcano plot depicting differential expression analysis between A549 WT and ΔUBR4 cells from bulk RNAseq analysis. Significantly upregulated and downregulated DEGs in ΔUBR4 cells are shown in red and blue, respectively (*P* < 0.05, log2-fold change > 1.6). DEGs highlighted in bold indicate the genes identified from both RNAseq and TMT-MS analyses. (*F*) Heat map showing DEPs from TMT-MS, grouped by cell cycle- and mitochondrial function-related BPs. WCLs from samples were labeled with 6-plex isobaric tags, analyzed by LC-MS^3^. The color key indicates the Z-score (red: upregulation; blue: downregulation). (*G*) IB analysis validating the TMT-MS results using WCLs from WT and ΔUBR4 cells. (*H*) Venn diagram representing the overlap of DEGs and DEPs upregulated in ΔUBR4 cells, with known genes in cell cycle and senescence pathways. (*I*) qRT-PCR showing a significant increase in *CDKN1A* mRNA levels in ΔUBR4 cells.

We first investigated whether UBR4-regulated protein homeostasis contributes to human cancer, based on previous findings suggesting dysregulated mitosis and cell growth in *UBR4*-mutant cells (27, 28). An unbiased analysis using The Cancer Genome Atlas (TCGA) datasets (31) revealed that *UBR4* mRNA expression was significantly higher in LUAD tumor samples (N = 510) than that in normal lung tissue samples (N = 59; *P* = 0.0014 from unpaired and *P* = 0.0085 from paired Student *t* tests; Fig. 1*A*). UBR4 protein levels, which previously correlated with *UBR4* mRNA levels, were also significantly elevated in LUAD tissues compared with those in normal controls (*SI Appendix*, Fig. S1 *A* and *B*). To better understand the functional signatures of UBR4 in LUAD, we performed gene set enrichment analysis (GSEA) using hallmark and gene ontology (GO) gene sets. GSEA of the hallmark gene sets identified that high *UBR4* levels were associated with cell cycle–related pathways such as G2/M checkpoints, whereas low *UBR4* levels were associated with mitochondrial functions, including OXPHOS (Fig. 1*B* and *SI Appendix*, Fig. S1 *C* and *D*). In addition, *UBR4* mRNA levels were positively correlated with *CDK6* (Spearman’s correlation; ρ = 0.03, *P* = 0.0001) and antigen identified by monoclonal antibody Ki67 (*MKI67*) (ρ = 0.347, *P* < 0.0001), and negatively correlated with *CDKN1A* (ρ = 0.120, *P* = 0.0072) in patients with LUAD (Fig. 1*C* and *SI Appendix*, Fig. S1*E*). Transcripts of various chemotaxis-stimulating factors, glutathione peroxidases, and mitochondrial proteins were also significantly correlated with *UBR4* expression (*SI Appendix*, Fig. S1*D*). Similarly, GO pathway enrichment using the DAVID database (32) revealed that *UBR4* in LUAD tumors was strongly associated with cell cycle regulation and mitochondrial respiration (Fig. 1*D*). These findings indicate a pathophysiological association between UBR4 and LUAD.

To investigate the oncogenic significance of UBR4 at the cellular level, we used the CRISPR/Cas9 system to knock out *UBR4* (ΔUBR4) in human adenocarcinoma lung cancer (A549) cells. RNA sequencing (RNAseq) identified 761 upregulated and 579 downregulated differentially expressed genes (DEGs; fold change > 1.6 and < –1.6, respectively; *P* < 0.05) in ΔUBR4 cells compared to those in WT cells (Fig. 1*E*). Many downregulated DEGs were OXPHOS components and assembly factors (*COA4/7*, *COX7B*, and *COX15/17*), as well as mitochondrial trafficking-related genes (*ARMCX3* and *MPV17L*) (Fig. 1*E*). Upregulated DEGs, including *CDKN1A*, *CXCL1/3/5/8*, and *SERPINE1/2*, were mostly involved in cell cycle regulation and senescence. Next, tandem mass tag-mass spectrometry (TMT-MS) was employed to quantitatively investigate global proteomic changes after UBR4 depletion. Out of the 9,695 proteins identified, the expression of 702 were significantly downregulated and 654 were upregulated in ΔUBR4 cells, based on a 1.5-fold change threshold with *P* < 0.05 (*SI Appendix*, Fig. S2*A*). When we compared these differentially expressed proteins (DEPs) with the RNAseq results, we found that upregulated DEPs in ΔUBR4 cells were primarily associated with cellular senescence and oxidative stress responses, whereas downregulated DEPs were involved in cell cycle progression and the OXPHOS pathway (Fig. 1*F*).

The proteomic changes were validated by immunoblotting (IB) of endogenous proteins. Consistent with the TMT-MS results, CDKN1A levels were much higher in ΔUBR4 cells than in WT A549 cells (Fig. 1*G*). The CDKN1A expression was consistently elevated across other ΔUBR4 clones (*SI Appendix*, Fig. S2*B*), substantiating the role of UBR4 in cell cycle regulation. In contrast, the levels of other cell cycle regulators (such as CDK2, CDK6, and CDKN2A) and the mitochondrial biogenesis regulator PPARGC1/PGC1 were significantly reduced. Similar consequences of *UBR4* knockout were observed not only in the A549 cell line (KRAS mutant, TP53 WT) but also in the H1975 (KRAS WT, EGFR/TP53 mutant), H23 (KRAS mutant, TP53 mutant), and murine LUAD LLC1 (KRAS1 mutant, TP53 WT) cell lines (*SI Appendix*, Fig. S2 *C*–*E*). Looking at the mutation states, such as TP53 and EGFR of LUAD cases, a significant enrichment of cell cycle- and mitochondrial function-related pathways was found in most samples, suggesting that UBR4’s pathological role is independent of specific oncogenic mutations in LUAD and in other lung cancer types as well (*SI Appendix*, Fig. S2 *F* and *G*). ClueGO-based enrichment further revealed that many upregulated DEPs were involved in functional protein networks, such as the mitotic cell cycle, DNA replication, and chromosome organization (*SI Appendix*, Fig. S2 *H* and *I*). Among the 761 upregulated DEGs in ΔUBR4 cells from RNAseq, 267 genes (35.1%) overlapped with proteins elevated in the TMT-MS analysis (*SI Appendix*, Fig. S2*J*). Many upregulated DEGs and DEPs in ΔUBR4 cells were involved in the cell cycle and senescence, and CDKN1A was located at the major intersections of multiple gene sets (Fig. 1*H*). We initially postulated that CDKN1A might be a direct target of UBR4; however, qRT-PCR indicated that *CDKN1A* mRNA levels increased while its proteasomal degradation at the posttranslational stage was actively processed even in cells lacking UBR4 (Fig. 1*I* and *SI Appendix*, Fig. S2 *K* and *L*). These findings suggest that the global transcriptome and proteome changes observed in ΔUBR4 cells are more likely to be driven by modified cellular energetics than by direct stabilization of cell cycle regulators.

### UBR4 Deficiency Drives Cell Cycle Arrest and Cellular Senescence.

**Fig. 2.**
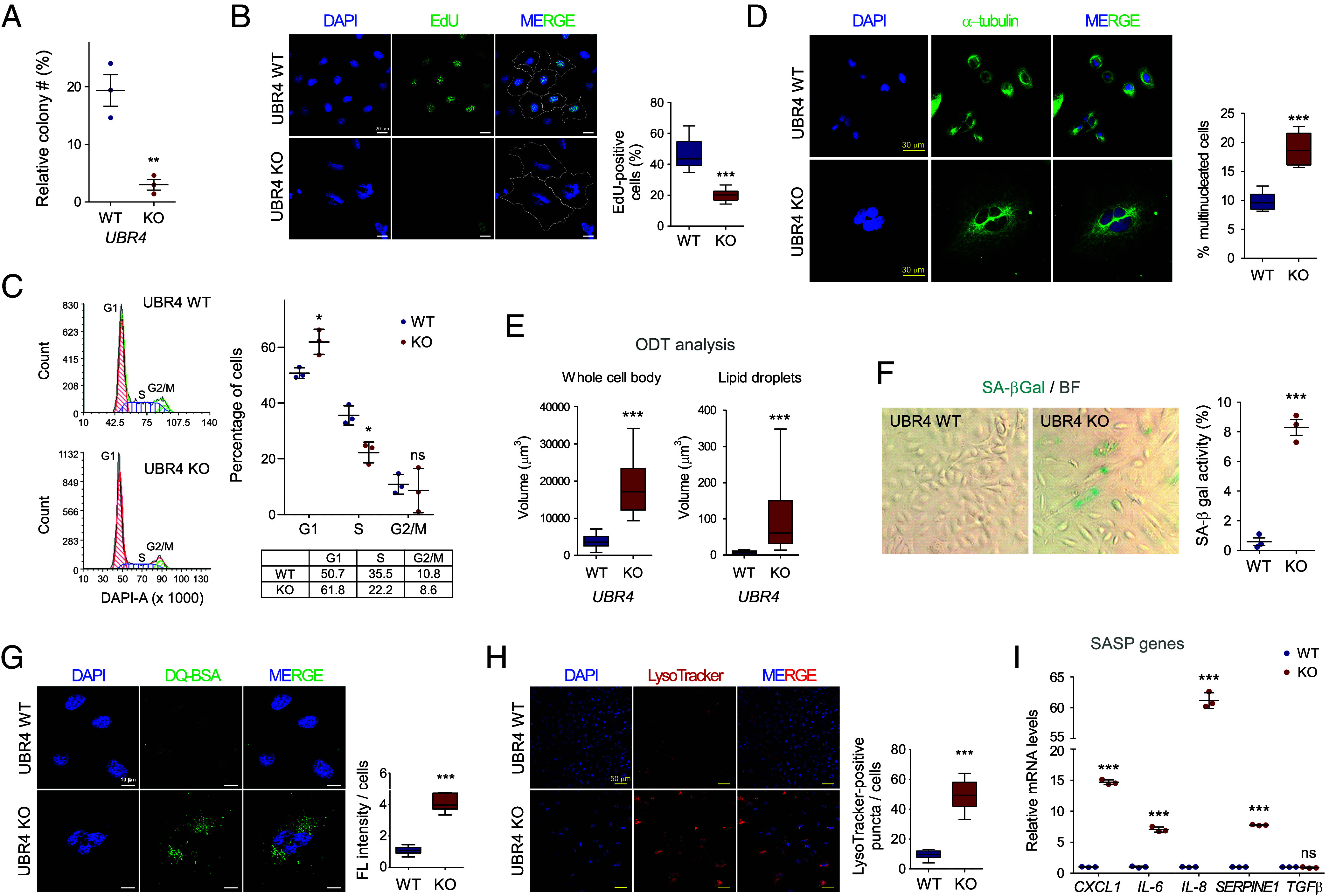
Impact of *UBR4* deficiency on senescence phenotypes in cultured LUAD cells. (*A*) Relative colony formation rates in A549 WT and ΔUBR4 measured by crystal violet staining after low-density seeding. Dot plots with SD error bars from three independent experiments. (*B*) As in (*A*), except that an EdU incorporation assay was performed. (*Left*) Representative images display the proliferation of cells treated with EdU for 2 h, visualized under a confocal fluorescence microscope (green). (*Right*) Quantification of the percentage of EdU/DAPI-double-positive cells from ~5,000 cells, presented as box (interquartile ranges) and whisker plots (min to max). Individual cell surfaces are displayed in dotted lines. (Scale bar: 20 μm.) (*C*) Flow cytometry-based cell cycle analysis quantifying DNA content with DAPI staining. The proportions of WT and ΔUBR4 cells at each cell cycle phase based on flow cytometry histograms are summarized in the table. Values are shown in dot plots. (*D*) Representative images of WT and ΔUBR4 cells with IFS using anti-α-tubulin (green) antibodies with DAPI counterstaining. The percentage of cells with multiple nuclei was counted and presented as a box and whisker plot from three independent experiments with ∼200 cells. (*E*) Optical diffraction tomograms of WT and ΔUBR4 cells were taken using Tomocube microscopy, and volumes of whole-cell bodies and lipid droplets were quantified using the built-in software. (*F*) Representative images and quantification of enzymatic staining (pH 6.0) of senescence-associated β-galactosidase (SA-β-gal) in A549 WT and ΔUBR4 cells. Dot plots showing percentages of SA-β-gal positive cells. BF, bright field. (*G*) Lysosomal cargo delivery was monitored by dequenching of DQ-Green BSA fluorescence (FL; 10 μg/mL for 1 h) and DAPI counterstaining. Representative images and average DQ-Green positive intensities are presented as a box and whisker plot from three independent experiments. (Scale bar, 10 μm.) (*H*) Lysosomal activity in WT and ΔUBR4 cells was monitored by staining with LysoTracker (75 nM for 1 h). (Scale bars are 50 μm.) (*I*) Secreted human SASP factors from WT and ΔUBR4 cells were analyzed with the bead-based Luminex multiplex assay. ***P < 0.001 from unpaired, two-tailed Student’s *t* tests (N = 3).

While conducting CRISPR/Cas9-mediated *UBR4* deletion, we observed that the proliferation of the ΔUBR4 cells was markedly slower than that of parental A549 cells, which was initially confirmed by comparing their growth rates and colony numbers (Fig. 2*A* and *SI Appendix*, Fig. S3 *A* and *B*). When cells were exposed to thymidine analogs, such as 5-ethynyl-2-deoxyuridine (EdU) and 5-bromo-2-deoxyuridine (BrdU), and their incorporation into the S phase was quantified with immunofluorescence staining (IFS) and ELISA, respectively, we found that the proportion of either EdU- or BrdU-positive ΔUBR4 cells was significantly lower than that of WT cells (Fig. 2*B* and *SI Appendix*, Fig. S3*C*). Flow cytometric analysis revealed that the reduced proliferation of ΔUBR4 cells was associated with a greater population of cells arrested at the G1–S transition (Fig. 2*C*). The percentage of ΔUBR4 cells in G1 and S phases were 61.9 ± 4.5% and 22.2 ± 3.7%, respectively, compared to 50.7 ± 1.9% and 35.5 ± 3.4% in WT cells. As an independent measurement, we performed the MTT assay, which measures the metabolic function of intracellular mitochondria based on succinate dehydrogenase activity and observed a significant cell growth delay in response to UBR4 knockout (*SI Appendix*, Fig. S3*D*). The mechanism of UBR4 deletion-induced proliferation arrest is expected to be linked to the changes in cell cycle regulatory proteins, such as significantly increased CDKN1A in ΔUBR4 cells.

Deletion of *UBR4* also resulted in substantial changes in cellular morphology; ΔUBR4 cells became enlarged and multinucleated (Fig. 2*D*). Quantifiable, label-free tomographic analysis, which essentially measures the different optical diffraction indices among cellular organelles, revealed that the ΔUBR4 cells had not only 5.7 times more total volume (18,461 ± 3,496 μm^3^ in ΔUBR4 vs. 3,837 ± 277 μm^3^ in WT cells) but also 15.2 times more cytoplasmic lipid droplets (average volume is 100.8 ± 50.5 μm^3^ vs. 6.6 ± 2.1 μm^3^) compared to WT cells (Fig. 2*E* and *SI Appendix*, Fig. S3*E*), potentially reflecting their cellular senescence state. Lipid droplet staining using BODIPY dyes yielded comparable results (*SI Appendix*, Fig. S3*F*). Upregulation of CDKN1A expression was observed after *UBR4* knockdown using siRNA although the effects were not as strong as those observed in ΔUBR4 cells (*SI Appendix*, Fig. S3*G*). Time-course analysis of *UBR4* knockdown also revealed significant cell cycle delay (*SI Appendix*, Fig. S3*H*). An add-back experiment using transient UBR4 overexpression in ΔUBR4 cells effectively reduced CDKN1A levels while increasing cell populations in the S and G2/M phases (*SI Appendix*, Fig. S3 *I* and *J*). Moreover, WT A549 cells showed a strong SA-β-gal activity and growth delay when treated with ΔUBR4-conditioned media (*SI Appendix*, Fig. S4 *A* and *B*). These results collectively indicate that the gross phenotypes observed in ΔUBR4 cells are not simple cellular artifacts of the knockout process but are directly related to UBR4 function.

The characteristics of ΔUBR4 cells described above are analogous to those of cells that have lost their proliferative capacity and become senescent (33). To further study this aspect, enzymatic staining of SA-β-gal was initially performed: As shown in Fig. 2*F*, the number of SA-β-gal-positive cells significantly increased among the ΔUBR4 cells, while there was virtually no signal in WT cells. We also found a considerable increase in lysosomal activity (measured by DQ-BSA and LysoTracker staining) in *UBR4*-null cells (Fig. 2 *G* and *H*), which further highlights their senescent characteristics. In contrast to cellular lysosomal activity, proteasome activity and levels remained comparable between WT and ΔUBR4 cells (*SI Appendix*, Fig. S4 *C* and *D*). We could not observe any significant apoptosis following the time-course knockdown of *UBR4* under normal conditions (*SI Appendix*, Fig. S4*E*). The expression of many SASP genes encoding proinflammatory cytokines, chemokines, proteases, and growth factors (34) and their secreted proteins were significantly upregulated in ΔUBR4 cells and their culture media, respectively (Fig. 2*I* and *SI Appendix*, Fig. S4*F*). Moreover, coculture of ΔUBR4 cells markedly delayed the growth of A549 EGFP cells, with the suppression becoming more pronounced as the proportion of ΔUBR4 cells increased (*SI Appendix*, Fig. S4*G*). These findings strongly support that UBR4 deficiency induces senescence through a paracrine mechanism driven by SASP factors. Because both phosphorylated TP53 and H2AX histone levels increased, senescence in ΔUBR4 cells is likely to be related to DNA damage (Fig. 1*G* and *SI Appendix*, Fig. S4*H*). Nevertheless, the cell proliferation rates were largely unaffected by *CDKN1A* knockdown (*SI Appendix*, Fig. S4*I*). These results suggest that *UBR4* depletion triggers cellular senescence, refuting the concept that cellular compensatory mechanisms in ΔUBR4 cells might drive phenotypic changes.

### UBR4-Deficient Lung Cancer Cells Showed Significant Mitochondrial Defects.

**Fig. 3.**
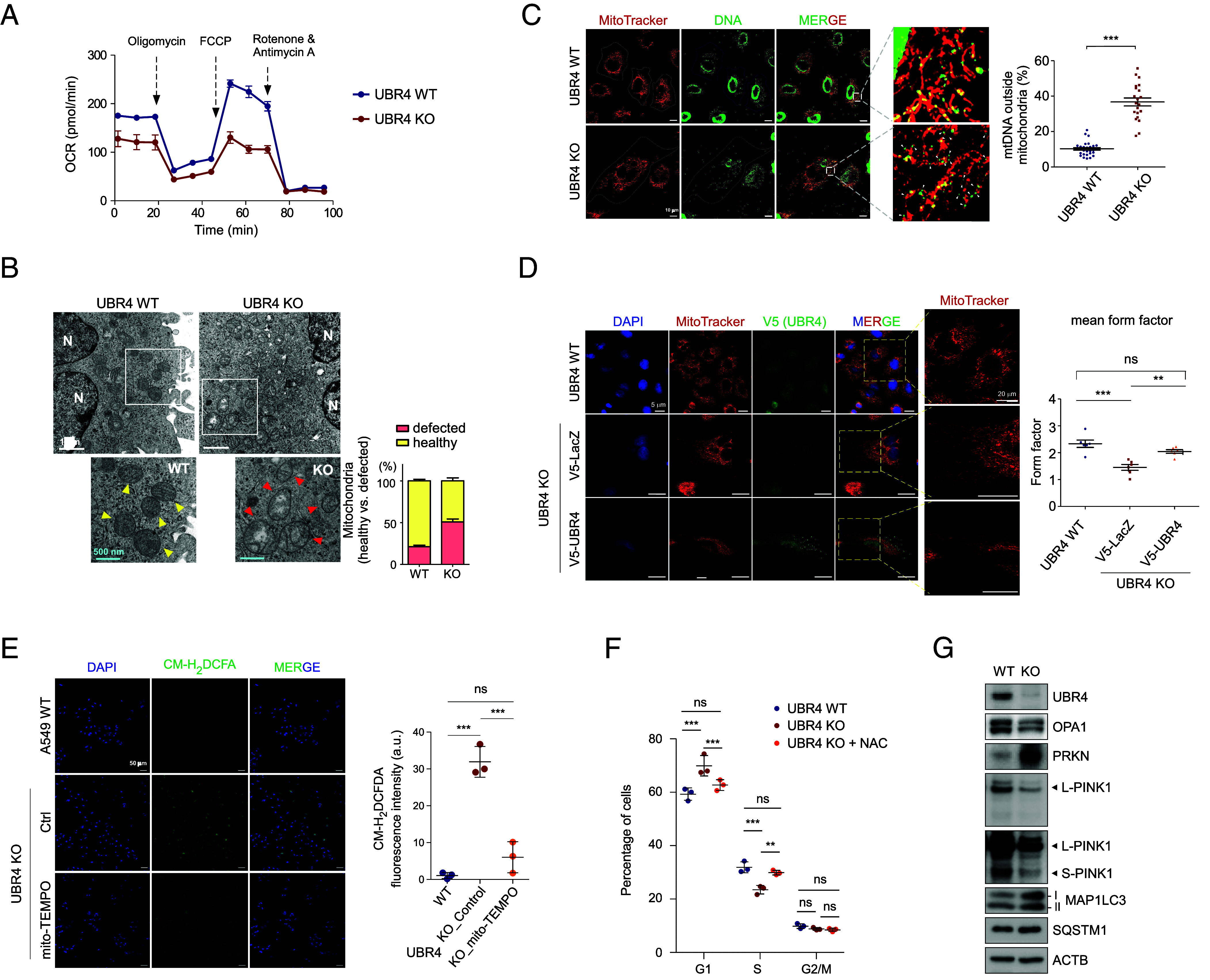
Impaired mitochondrial homeostasis and increased ROS in *UBR4*-null A549 cells. (*A*) A549 WT and ΔUBR4 cells were seeded in triplicates, allowed to adhere overnight, and treated with specified drugs (1 μM oligomycin, 0.5 μM carbonyl cyanide *p*-trifluoromethoxyphenylhydrazone [FCCP], and 0.5 μM antimycin A/0.5 μM rotenone, sequentially), following the standard Seahorse protocol to measure OCR. (*B*) Electron microscopy of WT and ΔUBR4 cells under normal conditions. Yellow and red arrowheads indicate healthy and damaged mitochondria (with enlarged morphology or disarrayed cristae), respectively. *Insets* are enlarged images of the enclosed area. White and blue scale bars represent 1 μm or 500 nm, respectively. N, nucleus. (*C*) Colocalization of mtDNA (anti-DNA staining in the cytosol; green) and mitochondria (MitoTracker Red-positive; red) was examined in WT and ΔUBR4 cells. Arrowheads indicate mtDNA outside mitochondria. (Scale bar: 10 µm.) Values are shown in dot plots from quantitative analysis of images (mean ± SEM). ***P < 0.001 (Student’s *t* test). (*D*) Representative image of mitochondrial morphology of WT and *UBR4* KO cells following transfection with V5-tagged LacZ or UBR4 for 36 h (*Left*). Quantitative data of the mitochondrial form factor of each cell (*Right*). **P < 0.01 and ***P < 0.001 from Bonferroni’s multiple comparison ANOVA test. (*E*) Representative confocal images (*Left*) of WT and ΔUBR4 treated with mitoTEMPO (100 nM) for 24 h, and CM-H_2_DCFDA (fluorogenic ROS probes; 10 μM for 1 h) fluorescent intensities normalized to DAPI signals (*Right*) are presented as dot plots with mean ± SD bars from three independent experiments (total 200 cells; ***P < 0.0001, One-way ANOVA followed by Tukey’s multiple comparison test to compare all pairs of means). (*F*) Cell cycle distribution of WT and ΔUBR4 cells before and after treating cells with 100 nM NAC for 30 h. **P < 0.01 and ***P < 0.001 from Bonferroni’s multiple comparison ANOVA test. ns, not significant. (*G*) Comparison of endogenous cytosolic and mitochondrial proteins, between WT and ΔUBR4 cells. WCLs were subjected to SDS-PAGE/IB using the indicated antibodies. Representative blots from two independent experiments are shown.

Given that alterations in cellular metabolism are inextricably related to energy-intensive processes such as the cell cycle, we reasoned that mitochondrial function could be responsible for senescence in *UBR4*-deficient cells and measured the mitochondrial O_2_ consumption rate (OCR) as a proxy for cellular OXPHOS capacity and energy production. We found that ΔUBR4 cells had a much lower OCR than WT cells (Fig. 3*A* and *SI Appendix*, Fig. S5*A*), indicating that severe mitochondrial dysfunction may underlie the progression of ROS-induced senescence in lung cancer cells (35, 36). Transmission electron microscopy revealed that ΔUBR4 cells had approximately 2.3 times more abnormal mitochondria than WT cells, which were distinguished by morphological characteristics such as swelling and disrupted cristae (Fig. 3*B*). Quantitative analysis of mitochondrial size after MitoTracker labeling revealed that ΔUBR4 cells had significantly enlarged mitochondria (*SI Appendix*, Fig. S5*B*), likely reflecting impaired mitochondrial dynamics. Furthermore, the increased release of mitochondrial DNA (mtDNA) into the cytosol underscored the mitochondrial defects associated with UBR4 loss (Fig. 3*C* and *SI Appendix*, Fig. S5*C*). Upon the add-back of UBR4, these mitochondrial abnormalities in ΔUBR4 cells were partially but significantly alleviated (Fig. 3*D*).

Considering that mitochondrial respiratory capacity serves as a reserve to protect cells from ROS (37), reduced OCR and imbalanced fission-fusion dynamics in ΔUBR4 cells may lead to oxidative stress and DNA damage. Indeed, we found that the cell-permeable ROS probe (CM-H_2_DCFDA) showed significantly higher levels of intracellular ROS in ΔUBR4 cells than in WT cells (Fig. 3*E*). To investigate whether mitochondrial dysfunction-mediated ROS production causes senescence, we treated the cells with mitoTEMPO, a mitochondria-specific antioxidant; the addition of mitoTEMPO significantly reduced the effect of *UBR4* deletion on elevated ROS levels (Fig. 3*E*), suggesting that ROS in ΔUBR4 cells primarily originate from the damaged mitochondria. In line with these findings, another potent antioxidant, *N*-acetylcysteine (NAC), moderately reversed the antiproliferative behavior of ΔUBR4 cells by promoting G1–S progression, significantly decreasing CDKN1A levels in ΔUBR4 cells (Fig. 3*F* and *SI Appendix*, Fig. S5*D*). Our findings collectively establish a causal link between UBR4 and cellular senescence, where mitochondrial homeostasis appears to be critically involved.

Although the underlying molecular pathways remain unclear, these findings suggest that ΔUBR4 cells experience oxidative stress because of defective mitochondrial homeostasis and implicate that UBR4 may give cells a survival advantage under mitochondrial stress. To understand the molecular basis of the accumulation of defective mitochondria in senescent ΔUBR4 cells, we first examined the PINK1/Parkin (PRKN) pathway and found that expression levels of PINK1 were significantly reduced in ΔUBR4 cells (Fig. 3*G*). In contrast, the expression of PRKN, which acts downstream of PINK1 to polyubiquitinate mitochondrial outer membrane proteins (38), was considerably increased. ΔUBR4 cells also showed considerably higher *PRKN*, microtubule-associated protein 1 light chain 3 (*MAP1LC3*), and *SQSTM1* mRNA levels than WT cells (*SI Appendix*, Fig. S5*E*). These findings implied a possible disruption in the mitophagy pathway; therefore, further assessment of overall mitophagy flux, as described below, was performed to identify the underlying mechanism.

### UBR4 Plays a Vital Role in the Clearance of Defective Mitochondria through Mitophagy.

**Fig. 4.**
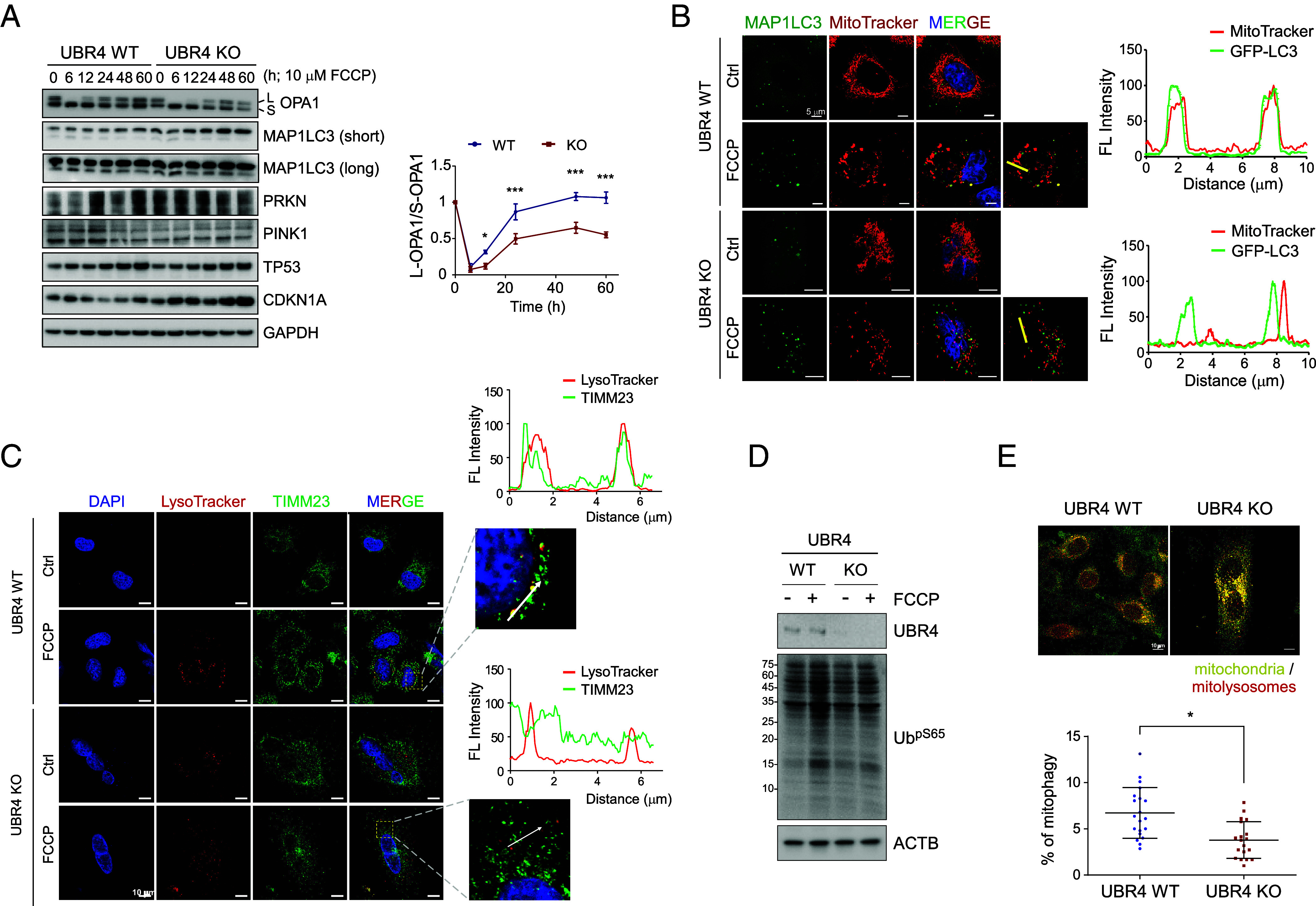
Dysfunction of autophagy-mediated mitochondrial quality control in *UBR4*-deficient cells under mitochondrial stress. (*A*) Time-course IB analysis (*Left*) and quantification (*Right*) of the proteolytic cleavage and recovery of OPA1. WT and ΔUBR4 cells were incubated with 10 μM FCCP or with vehicle (0.1% [v/v] DMSO) to block OXPHOS for the indicated periods. For each time point, the long (L) and short (S) forms of OPA1 bands were quantified and normalized to those of endogenous GAPDH. The L-OPA1:S-OPA1 ratios are presented as mean ± SD from three independent experiments. *P < 0.05, ***P < 0.001 from Bonferroni’s multiple comparison ANOVA tests). (*B*) Cells were transfected with GFP-MAP1LC3 for 24 h, treated with 10 μM FCCP for an additional 12 h, and incubated with 100 nM MitoTracker Red for 30 min. Representative confocal fluorescence (FL) images and profiles showing mitochondria (MitoTracker) and autophagosomes (MAP1LC3) fluorescence intensities across the yellow line are shown. (Scale bar: 5 μm.) (*C*) Similar to (*B*), except that IFS with anti-TIMM23/Tim23 antibodies (green) was used to localize mitochondria and LysoTracker Red DND-99 (100 nM for 1 h; red) was used to label lysosomes. Representative confocal fluorescence images and profiles showing mitochondria (TIMM3) and autolysosome (LysoTracker) fluorescence intensities across the yellow line are shown. (Scale bar: 10 μm.) (*D*) The level of phosphorylated ubiquitin at Ser65 was evaluated in WT and UBR4 KO cells with or without FCCP (10 μM for 12 h). (*E*) Detection of mitophagy using a pH-dependent mitochondria-targeted Keima (mito-Keima). Cells stably overexpressing mito-Keima were visualized using 458 nm (green, mitochondria at neutral pH) and 561 nm (red, mitochondria at acidic pH) laser lines and a 575 nm filter after treatment with 10 µM FCCP for 6 h. Representative images (*Left*) and quantification of mitophagy in the presence of FCCP are shown (means ± SD; *P < 0.1 from Bonferroni’s multiple comparison ANOVA). (Scale bar: 10 µm.)

To determine whether and how UBR4 affects the amplitude or duration of mitochondrial dynamics, we monitored the time-course cleavage of the inner mitochondrial protein OPA1, a mitochondrial dynamin-like GTPase after treatment with carbonyl cyanide 4-(trifluoromethoxy)phenylhydrazone (FCCP), which uncouples OXPHOS from ATP production. Damaged mitochondria with disrupted OXPHOS are known to cause full-length OPA1 cleavage and inactivate mitochondrial fusion (39). In ΔUBR4 cells, recovery of OPA1 cleavage was significantly slower than that in WT cells, suggesting that the delayed fission (Fig. 4*A*) may be linked to impaired mitochondrial turnover.

We observed substantial colocalization of mitochondria and autophagosomes in WT cells using live-cell fluorescence analysis, visualized with MitoTracker Red and GFP-tagged MAP1LC3, respectively (Fig. 4*B*). On the contrary, we consistently observed that ΔUBR4 cells had minimal colocalization with autophagosomes. Live-cell fluorescence analysis also revealed that, upon FCCP treatment, mitophagy was notably impaired in ΔUBR4 cells compared to that in WT cells. In WT cells, mitochondria showed substantial colocalization with autophagosomes (Fig. 4*B*). In sharp contrast, in ΔUBR4 cells, not only the colocalization of mitochondria with autophagosomes but also the transport of mitochondria to LysoTracker Red-labeled lysosomes was significantly reduced, indicating a marked defect in mitophagy (Fig. 4 *B* and *C*).

Furthermore, upon FCCP treatment, phospho-Ser65 ubiquitination levels were lower in ΔUBR4 cells than WT cells, suggesting a deficiency in ubiquitination that may hinder mitophagy initiation in the KO cells (Fig. 4*D*). To directly examine the impact of UBR4 deletion on mitophagy, we stably overexpressed the pH-sensitive tandem reporter mito-Keima, which quantitatively distinguishes free, cytoplasmic mitochondria from those within autolysosomes (40), via lentiviral transduction and concomitant selection. Confocal microscopic analysis revealed a significant increase in the number of WT cells containing mitolysosomes after FCCP treatment, whereas ΔUBR4 cells exhibited a much smaller increase (Fig. 4*E*). These results strongly indicate that UBR4 deletion impairs efficient mitophagy progression under mitochondrial stress.

### Deletion of *UBR4* Markedly Attenuated Tumor Growth in Mouse Xenograft Models.

**Fig. 5.**
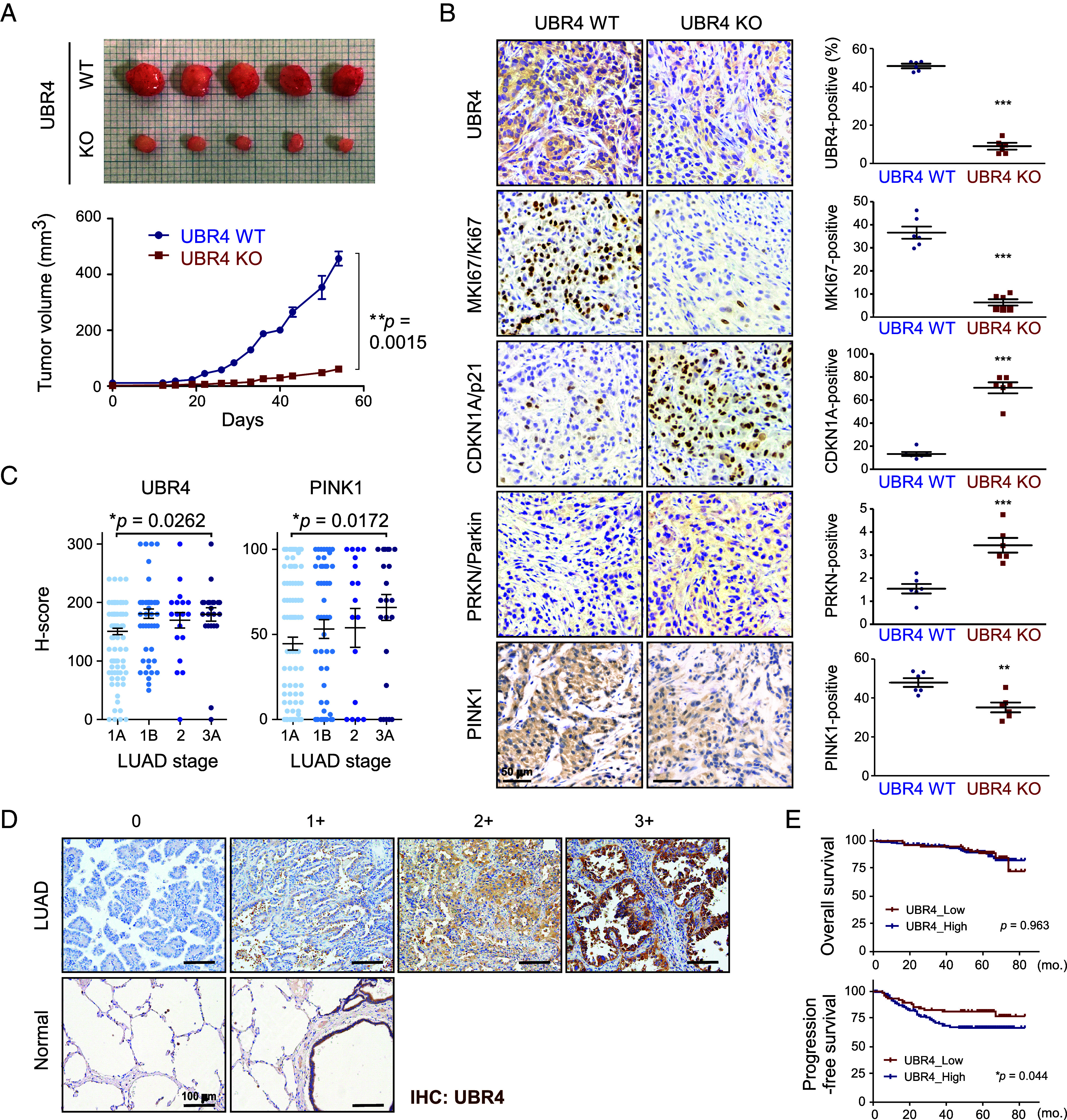
The necessity of UBR4 for the in vivo tumor growth and potential metastasis of LUAD. (*A*) Representative images of tumors (*Top*) formed from subcutaneous inoculation of 1 × 10^6^ WT and ΔUBR4 A549 cells into NSG mice on day 54. The graph (*below*) demonstrates the quantitative evaluation of average tumor volume over time. Data are presented as the mean ± SD (N = 6). Statistical analysis was performed by the Mann–Whitney *U* test. (*B*) IHC analysis of paraffin sections from tumor nodules, including UBR4, MKI67, CDKN1A/p21, PRKN/Parkin, and PINK1, along with hematoxylin-eosin staining. Representative images (*Left*) and quantitative analysis (*Right*) for each group are displayed, with statistical significance indicated by *P < 0.05, **P < 0.01 and ***P < 0.001 (Student’s *t* test). (*C*) UBR4 (*Left*) and PINK1 (*Right*) protein levels (mean ± SEM) among four stage subsets, determined by lung cancer pathologists. H-scores show significant differences between LUAD stages 1A and 3A (*P* = 0.0262 for UBR4 and 0.0172 for PINK1 by the *t* test with Welch’s correction). N = 123, 59, 22, and 26 for stages 1A, 1B, 2, and 3A, respectively. (*D*) Representative IHC images of human LUAD TMAs showing the increasing UBR4 staining intensity with disease stage scoring by the pathologist. (Scale bars, 100 µm.) (*E*) Spearman correlation analysis of UBR4 protein levels and overall survival (*Top*) or progression-free survival (*Bottom*). The cut-off values of UBR4_high and UBR4_low groups were defined by their median expression (H-score = 150). The Kaplan–Meier method with the log-rank test determined the *P-*values.

To demonstrate the consequences of UBR4 deletion and accompanying cellular changes in vivo, we employed a xenograft LUAD model. Six-week-old female NOD/SCID/IL-2γ-receptor null (NSG) mice were subcutaneously injected with equal quantities of A549 WT and ΔUBR4 cells, and tumor volumes and body weights were evaluated every 3 d over an 8-wk period. At day 54, we found that although WT cells formed readily visible tumors, ΔUBR4 cells exhibited markedly impaired tumorigenic capacity, resulting in significantly smaller tumors compared to WT controls (456.8 ± 57.0 mm^3^ in WT vs. 61.1 ± 21.2 mm^3^ in ΔUBR4 cells; *P* = 0.0015 from Mann–Whitney *U* test; Fig. 5*A*). In sharp contrast, no difference in body weights was observed between the experimental groups (*SI Appendix*, Fig. S6*A*).

To assess protein expression levels in xenografted tumors, we performed immunohistochemical (IHC) staining of cross-sections, which revealed strong negative correlations between UBR4 and CDKN1A (Fig. 5*B*). Strong expression of PRKN in ΔUBR4 tumors also mirrored the indispensable role of UBR4 in mitophagy. The decreased proliferation of *UBR4*-deficient tumors was evidenced by a significantly lower MKI67 expression. Furthermore, positive PINK1 staining was observed in fewer than 35.2% of ΔUBR4 tumors, compared to over 47.9% in WT tumors (*P* = 0.01, unpaired Student’s *t* test; Fig. 5*B*). While the levels of senescent (GL13-positive) cells were markedly increased in the ΔUBR4 xenografted tumors, the number of apoptotic (active caspase 3- or TUNEL-positive) cells was comparable to that observed in WT tumors (*SI Appendix*, Fig. S6 *B*–*D*). These results are highly consistent with our observations that UBR4 expression is positively linked to human LUAD tissues, and its ablation causes cell cycle defects and senescence in cultured lung cancer cells.

To evaluate UBR4 and PINK1 (as a mitophagy marker) protein levels in human lung cancer, IHC staining was performed on a TMA of human LUAD samples (N = 250 vs. normal tissue N = 10; *SI Appendix*, Tables S1 and S2). We observed a consistent trend of increased IHC staining intensity correlated with larger tumor sizes and advanced LUAD stages in both UBR4 (H-scores 179.6 ± 11.29 in Stage 3A [N = 26] vs. 150.6 ± 5.49 in Stage 1A [N = 123]; *P* = 0.0262) and PINK1 (H-scores 65.77 ± 7.61 in Stage 3A vs. 44.60 ± 3.78 in Stage 1A; *P* = 0.0172 from *t* test with Welch’s correction) (Fig. 5 *C* and *D* and *SI Appendix*, Fig. S6*E*). In a similar manner, the protein levels (H-scores) of UBR4 and PINK1 in tumors showed a significant positive correlation (Spearman’s correlation; r = 0.496, *P* < 0.001; *SI Appendix*, Fig. S6*F*). Finally, we found that UBR4, not PINK1, expression was significantly negatively associated with progression-free survival (*P* = 0.044 from the Kaplan–Meier log-rank test) but showed no significant association with overall survival rates (Fig. 5*E* and *SI Appendix*, Fig. S6*G*).

These data provide strong clinical evidence for the pathological significance of UBR4 and mitophagy in human LUAD, emphasizing its tumor-promoting characteristics. Together, our data clearly demonstrate the tumor-promoting characteristics of UBR4 in both lung cancer cells and mouse models, indicating that its deletion reduces tumor growth and induces senescence. UBR4 inhibitors have the potential to be effective antitumor drugs that suppress the proliferation of LUAD cells either alone or in combination with existing treatments.

## Discussion

The pathophysiological role of UBR4 remains largely unclear despite its recent identification in the integrated stress response (SIFI) pathway (19, 28). In the SIFI complex, UBR4 mitigates chronic stress responses by targeting cytosolic mitochondrial proteins, which often trigger apoptotic pathways. We identified a functionally analogous but mechanistically distinct role of UBR4 in coordinating cellular fate via senescence, apoptosis, and oncogenesis (*SI Appendix*, Fig. S6*H*). Among previous studies on cellular and organismal phenotypes after *UBR4* knockout, what initially drew our attention was a marked delay in the cell cycle (27). We observed significantly elevated *UBR4* and reduced *CDKN1A* expression in human LUAD tumor tissues from clinical databases. *UBR4* deletion resulted in cell cycle arrest, a hypersecretory and proinflammatory phenotype, and paracrine senescence induction in cultured lung cancer cells, along with a substantial delay in ΔUBR4 LUAD tumor growth in an in vivo mouse xenograft model. Our findings indicate a direct causative effect of UBR4 on senescence, though further investigation is needed to clarify the direct signaling pathway(s) responsible for these effects. *UBR4*-deficient cells displayed an imbalance in mitochondrial dynamics, favoring fusion over fission, alongside severe mitochondrial damage and reduced mitophagic clearance of faulty or superfluous mitochondria. Our data suggest that UBR4 acts as an agonistic upstream effector, promoting global mitochondrial remodeling via enhanced mitophagy. Thus, we propose that the UBR4–mitophagy axis serves as a critical triage point for the reciprocal coordination of senescence and apoptosis, two mutually exclusive cellular responses, under chronic stress conditions, particularly under mitochondrial stress conditions. Whether UBR4 exhibits general oncogenic properties or cancer-type specificity, as seen in LUAD, remains to be determined.

Although our data support a model where UBR4-mediated mitophagy under mitochondrial stress improves the metabolic adaptation of LUAD tumors, further studies are required to identify the UBR4 substrates that directly contribute to downstream regulatory pathways. Key components of mitophagy such as PINK1, are known to be targeted by UBR1 and UBR2 rather than UBR4 (41). Additionally, the UBR box in UBR4 is not necessary for SIFI-mediated substrate ubiquitination, which primarily recognizes mature (processed) mitochondrial proteins (28). These results implicate that UBR4, a unique E3 Ub ligase with a “hemiRING” domain, detects “converging degrons” only after the irreversible removal of mitochondrial-targeting sequences and subsequent conformational changes. This degron-generating process resembles the biochemical production of primary N-degrons via single or multistep enzymatic modifications (42, 43). Active endopeptidases may contribute this integrated stress response mechanism, underlying the molecular pathology of LUAD. A significant gap remains in understanding the mechanistic and physiological functions of UBR4, one of the largest single-subunit E3 Ub ligases, with a 574 kDa mass. For instance, studies have revealed that UBR1, UBR2, and UBR4 possess autoubiquitination activity and that UBR4 levels increase in aged fruit flies and mice (44, 45). This increase in UBR4 expression with age may be directly related to the age-associated upregulation of mitophagy (46, 47). The precise sequence of biochemical events, the impact of mitochondrial stress on UBR4 autoubiquitination, and their substrate specificity are yet to be elucidated.

The present study also indicates that UBR4 loss causes cell cycle arrest as an initial response to oncogenic stimuli, potentially serving as a tumor suppression mechanism. Conversely, UBR4 appears to facilitate metabolic reprogramming in cells exposed to chronic inflammatory stimuli or DNA-damaging agents. UBR4 seems crucial for compensatory responses to chronic mitochondrial dysfunction via mitophagy. In ΔUBR4 cells, senescence appears to be mediated via the TP53–CDKN1A pathways rather than the CDKN2A–Rb axis, bypassing apoptotic responses. Nonetheless, CDKN1A is unlikely to be the sole driver of the complex changes associated with UBR4-mediated senescence (48), indicating a close association between senescence traits and dysfunctional mitophagy. It is possible that the UBR4 functions upstream of the TP53–CDKN1A pathway and may have different effectors regulating the senescence process. We also found that antioxidants effectively inhibit *UBR4* knockout-mediated senescence, consistent with earlier studies showing that cellular ROS levels are critical in CDKN1A-mediated cell fate determination (49). Mitochondrial ROS can damage nuclear DNA, initiating the DNA damage responses that lead to senescence. Furthermore, abnormal mitochondrial output can provide a growth disadvantage to cancer cells. Therefore, it seems that cells interpret UBR4 inactivation as a survival signal under persistent mitochondrial or ROS stress and that UBR4 levels could be used as prognostic markers and therapeutic targets for LUAD.

Mitochondrial dysfunction in ΔUBR4 cells directly induces senescence, accompanied by excessive ROS, decreased OXPHOS, and elevated DNA damage. Redox imbalance and increased ROS may activate NF-κB signaling and the inflammatory pathway in SASPs. Although these relationships are complex, our findings collectively suggest that UBR4 actively functions as a sensor of dynamic environmental changes, maintaining mitochondrial dynamics and metabolic pathways in tumor cells. In this model, UBR4 inactivation may protect cells from DNA damage and minimize apoptotic cell death as well as the transition to tumorigenesis under prolonged adverse conditions. It is possible that the tumor-promoting ability of the UBR4–mitophagy axis is suppressed under normal conditions by endogenous antagonists or inhibitory proteins but activated by cellular endoproteolytic mechanisms. Therefore, understanding this tonic inhibitory mechanism could lead to novel LUAD therapeutic targets. This hypothesis has broader implications for other chronic stress–related pathologies, as senescent cells can prevent the proliferation of premalignant or malignant cells through paracrine effectors, such as SASPs. Single-cell genomics and spatial multiomics will provide more rigorous quantitative assessments of UBR4-mediated senescence and its interplay with the tumor microenvironment, potentially indicating the clinical relevance of UBR4-targeting strategies in treating LUAD and other cancers with dysregulated mitochondrial homeostasis.

## Material and Methods

### Design of sgRNAs and Generation of the ΔUBR4 Cell Line.

sgRNA sequences targeting exonic regions of the UBR4 gene (RefSeq: NC_000001.11) were identified using CRISPR RGEN Tools. The selected sgRNA sequences, 5′-AGGATGTCTCTGACTCGGA-3′, targets exon 11 of *UBR4*. For cloning, two oligonucleotides containing spacer sequences, 5′-caccgAGGATGTCTCTGACTCGGA-3′ and 5′-aaacTCCGAGTCAGAGACATCCTc-3′, were annealed to form double-stranded DNA fragments with compatible overhangs at BsaI sites. These oligonucleotides were ligated into BsaI-digested pRG2 (Addgene, 104174) using T4 DNA ligase. To generate the ΔUBR4 cell line, 2 × 10^5^ A549 cells were seeded in a 24-well plate. After 24 h, the cells were transfected with 0.75 µg of p3s-pSpCas9 (Addgene, 104171) and 1 µg of sgRNA expression plasmid using Lipofectamine 3000. For clonal selection, cells were seeded in 96-well culture plates at a low density of 0.5 cells/well), and knockout efficiency was confirmed by targeted next-generation sequencing (NGS).

### Measurement of Lysosomal Acidity and Proteolytic Activity.

To evaluate lysosomal acidity and proteolytic activity, both WT and ΔUBR4 cells were cultured until reaching ~30% confluency. Cells were then stained with LysoTracker Red DND-99 (final concentration 75 nM; TFS, L7528) or DQ-BSA Green (final 10 µg/mL; TFS, D12050) for 1 h at 37 °C to label active acidic lysosomal compartments and assess lysosomal degradation in cells, respectively. Following staining, the cells were washed with PBS and mounted with Fluoroshield mounting medium containing DAPI (Abcam, ab104139). The fluorescence microscopy images were captured and analyzed using ImageJ for graphing and statistical analysis.

### SA-β-Gal Staining.

To assess SA-β-gal activity, cells were seeded in six-well plates, cultured until reaching 70 to 80% confluency, fixed in 4% paraformaldehyde (PFA) for 10 min, and stained with the Senescence β-Galactosidase Staining Kit (CST, 9860.) Following staining, cells were incubated at 37 °C without CO_2_. Quantification of SA-β-gal positive cells involved counting blue cells in randomly selected fields from each sample, with at least 200 cells counted per sample.

### Measurement of Cellular ROS Levels.

Intracellular ROS levels were assessed by treating cells with permeable CM-H_2_DCF-DA (TFS, D399) and analyzing the images obtained using a fluorescence microscope (excitation and emission 495/527 nm). Briefly, cells grown on glass slides were exposed to 10 μM CM-H2DCF-DA for 10 min at 37 °C. After two washes with PBS, cells were fixed with 4% PFA (pH 7.4) for 30 min, washed again with PBS, and mounted using coverslips and mounting medium containing DAPI.

### Assessing Proteasome Activity Using Fluorogenic Peptide Substrates.

To monitor the proteolytic activity of cellular proteasomes, hydrolysis of fluorogenic peptide substrates, 7-amino-4-methylcoumarin (suc-LLVY-AMC; Bachem), was quantified. Briefly, the assay involved incubating 10 µg of wholecell lysates (WCLs) with 12.5 μM of suc-LLVY-AMC in proteasome activity assay buffer (50 mM Tris-HCl [pH 7.5], 1 mg/mL BSA, 1 mM EDTA, 1 mM ATP, and 1 mM DTT) (50). The AMC fluorescence of liberated AMC was measured in a black 96-well plate using Infinity 200 PRO (Tecan).

### Measurement of OCR.

The OCR was measured using a Seahorse XFe24 Analyzer and related reagents from Agilent unless otherwise specified. Cells were plated at ~2.5 × 10^5^ cells per well in a Seahorse XF24 Cell Culture Microplate and cultured under standard conditions (37 °C, 5% CO_2_) in RPMI until reaching 70 to 80% confluency. Cells were treated with Seahorse XF medium supplemented with 2 mM l-glutamine and 10 mM d-glucose (final volume of 555 μL), incubated at 37 °C in a 0% CO_2_ chamber for 1 h, and transferred to a Seahorse XFe24 Analyzer. Cellular respiration was evaluated using the Cell Mito Stress Test (Agilent, 103015-100) in triplicate, following the manufacturer’s instructions. OCR data were normalized to cell number per well using a bicinchoninic acid assay (GenDEPOT, P8100-050) and are presented as means ± SEM.

### mtDNA Release and Replication.

To evaluate mtDNA release into the cytosol, DNA was purified from cytosolic fractions using a Puregene DNA Isolation Kit (Qiagen, 158043), and qRT-PCR was performed to assess ATP8A levels in the cytosol, providing a measure of mtDNA release. GAPDH served as a normalization control. To visualize cytoplasmic mtDNA using an anti-DNA antibody and MitoTracker, cells were seeded into 24-well plates and cultured at 37 °C for 24 h until reaching ~50% confluency. Following fixation and permeabilization, cells were incubated with anti-DNA antibody. For imaging and quantification of cytoplasmic mtDNA, cells were imaged using a Zeiss LSM980 Airyscan2 confocal laser scanning microscope. To quantify cytosolic mtDNA copies, ~20 total cells were randomly quantified using Fiji software.

### Tumor Xenograft Models.

Mice were housed in a facility fully accredited by the Institutional Animal Care and Use Committee of Seoul National University. Female NSG mice, aged 6 to 8 wk (Jackson Laboratory), were subcutaneously implanted with either WT or ΔUBR4 cells (1 × 10^6^ cells) on the right flank with a 26-gauge syringe. Tumor size was monitored every 3 d using calipers, and body weight was measured twice weekly. Tumor volume was calculated using the formula: [length/2] × [width] × [width]. Mice were killed 8 wk after implantation, and tumors were harvested for subsequent IHC analyses.

### TMA Analysis of Specimens from LUAD Patients.

For TMA, tissues were collected from 250 patients who underwent surgery for LUAD at Seoul National University Hospital from 2016 to 2018. Collected samples were fixed in 10% neutral formalin for 24 h at room temperature before being embedded in paraffin. TMAs were constructed from 2-mm diameter cores derived from representative tumor areas of formalin-fixed paraffin-embedded (FFPE) tissue blocks. FFPE blocks of normal lung tissue were also collected from resected specimens of 10 LUAD patients. No patients had received chemotherapy before surgery. This study followed recommendations of the World Medical Association Declaration of Helsinki recommendations and was approved by the Institutional Review Board of SNUH (H-1404-100-572). All human samples were deidentified before use.

ICH for PINK1 and UBR4 was performed with the aforementioned antibodies and conditions, using a Bond-Max Autostainer (Leica Microsystems). PINK1 and UBR4 expression were evaluated in terms of staining intensity and the proportion of stained tumor cells. IHC staining was scored as 0 (no staining), 1+ (weak staining), 2+ (moderate staining), or 3+ (strong staining), based on the intensity of cytoplasmic expression, and the proportion (%) of tumor cells with each score was determined. The H-score was calculated by multiplying the intensity score by the percentage of positive tumor cells and ranged from 0 to 300. The cut-off value was defined as the median expression for each antibody (Median H-scores were 60 for PINK1 and 150 for UBR4).

## Supplementary Material

Appendix 01 (PDF)

## Data Availability

The mass spectrometry proteomics data have been deposited in the ProteomeXchange Consortium via the PRIDE partner repository (PXD051417) (51). All other data are included in the manuscript and/or *SI Appendix*.
