# Supplementary material for: Tumor-promoting UBR4 coordinates impaired mitophagy–associated senescence and lung adenocarcinoma pathogenesis
Source: Proc Natl Acad Sci U S A. 2025 Jun 18;122(25):e2425015122. doi: 10.1073/pnas.2425015122 (PMC12207436; doi:10.1073/pnas.2425015122)
Supplement: Supplementary file 1 — Appendix 01 (PDF) [file pnas.2425015122.sapp.pdf]

PNAS
www.pnas.org

Supplementary Information for

Tumor-Promoting UBR4 Coordinates Impaired Mitophagy-Associated Senescence and Lung Adenocarcinoma Pathogenesis

Dawon Jeong, Seo Hyeong Park, Jiwon Kim, Hyeyoon Kim, Yejin Jang, Jaemoon Koh, Yoon Kyung Jeon, Takafumi Tasaki, Yong Tae Kwon, Dohyun Han, Sung-Yup Cho, Min Jae Lee*

*Min Jae Lee
Email: minjlee@snu.ac.kr

This PDF file includes:

Supplementary Figures 1 to 6
Supplementary Tables
Supplementary Methods
Supplementary References

SUPPLEMENTARY FIGURES

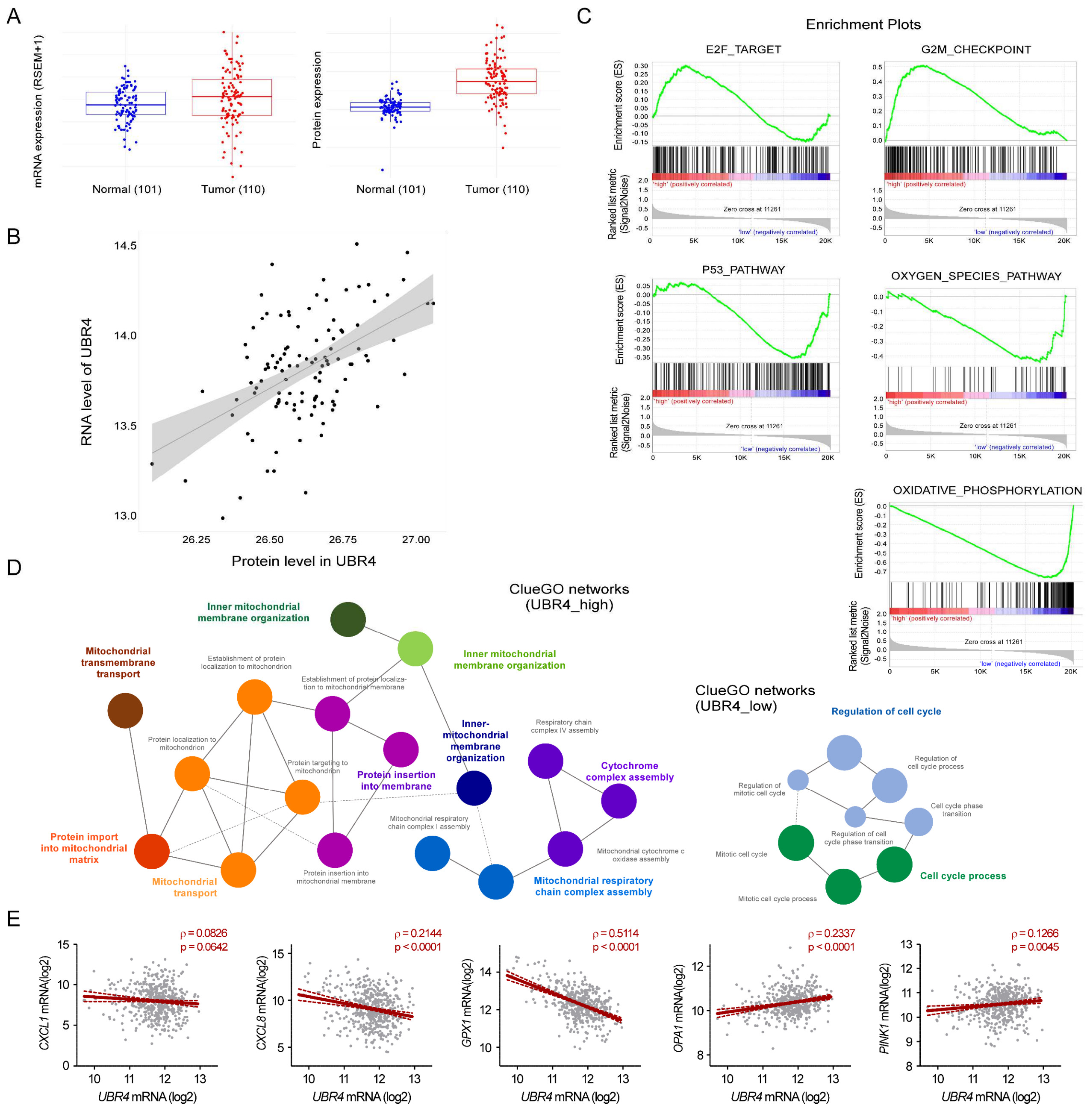

Supplementary Figure 1. Overall protein and transcriptome profile associated with the *UBR4* expression levels in patients with lung adenocarcinoma (LUAD)

(**A**) Comparison of UBR4 mRNA (*left*) and protein (*right*) expression levels between normal and tumor LUAD tissues, based on analysis from the Clinical Proteomic Tumor Analysis Consortium (CPTAC) database. Log2-transformed normalized RNAseq expectation maximization (RSEM) values (p = 0.03433; *left*) and protein intensity values (p = 1.662e-23;

right) are shown. Statistical significance was determined using the Wilcoxon rank-sum test. N = 101 for normal controls and N = 110 for LUAD samples. (**B**) Scatter plot showing the positive correlation between UBR4 mRNA and protein expression in LUAD samples from the CPTAC pan-cancer proteogenomic data (N = 110). The x-axis represents the log2 intensity of protein expression levels, and the y-axis represents normalized RSEM values that were log2-transformed. The solid line depicts the regression curve, and gray areas indicate 95% confidence intervals (N = 110; ρ = 0.1 and p = 1.1e-0.5 from Spearman's correlation analysis). (**C**) Enrichment plots depicting significant correlations between the gene sets associated with TCGA-derived LUAD patients. *Top*, UBR4_high, including E2F and G2M checkpoints. *Bottom*, upregulated pathways in UBR4_low samples include TP53 signaling, reactive oxygen species, and oxidative phosphorylation. (**D**) Interaction analysis among GSEA-enriched genes using the ClueGO platform ($p < 0.05$, kappa score = 0.7). Genes associated with the same GO term (biological process; BP) are represented by the identical node/letter color. The circle size depicts the group p-values < 0.05, < 0.01, or < 0.001, from the smallest to the biggest. Dashed lines indicate weak (indirect) connectivity. (**E**) Spearman correlation analysis of *UBR4* expression and other cell cycle (*CXCL1*, *CXCL8*, and *GPX1*) and mitochondrial dynamics (*OPA1* and *PINK1*)-related mRNA in LUAD patients from cBioportal. Solid and dashed lines depict regression lines and 95% confidence intervals, respectively. These data complement Figures 1A – 1D.

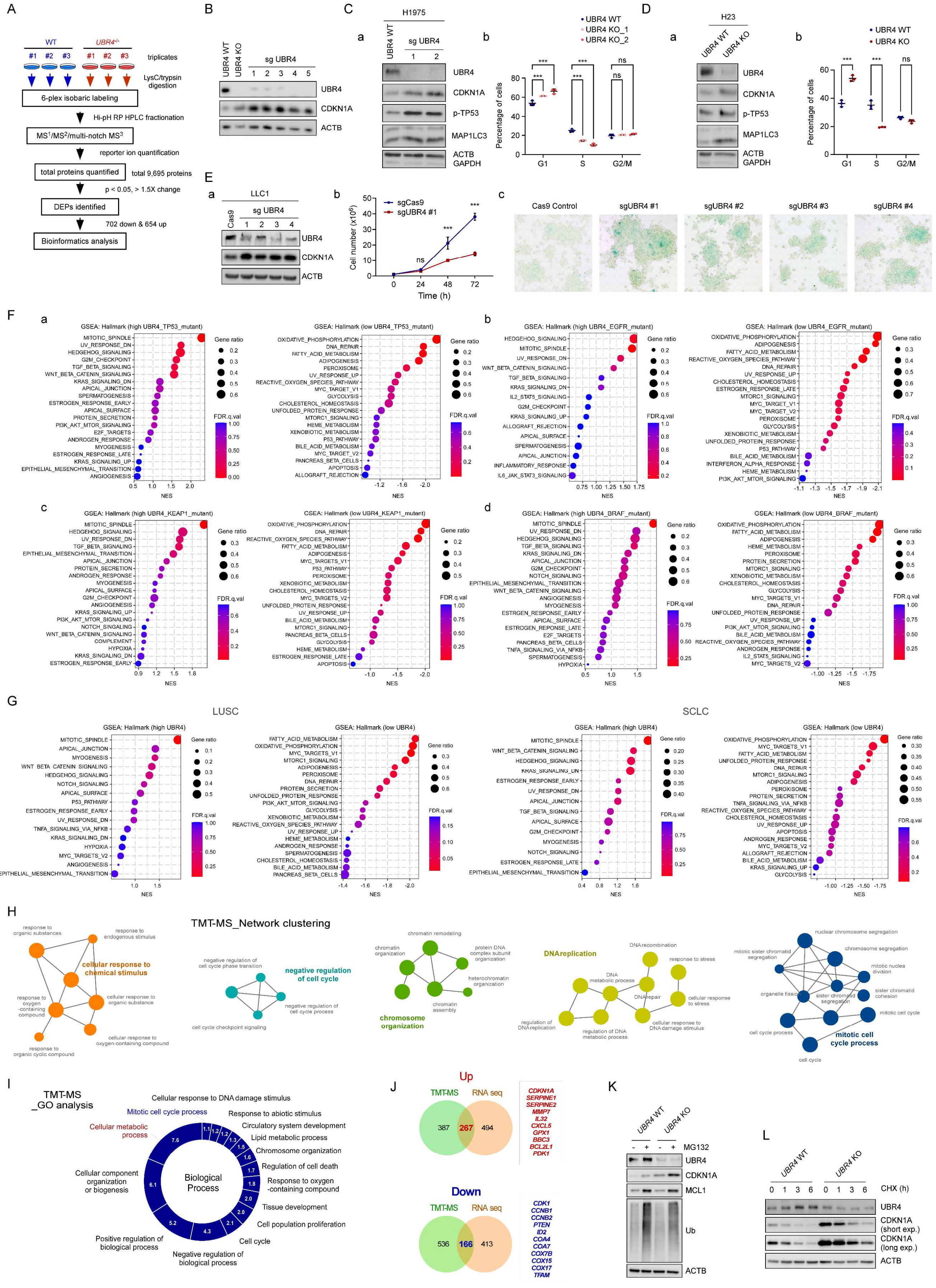

Supplementary Figure 2. Comparative analysis between the proteomes from A549 WT and *UBR4* KO cells

(**A**) LC-MS^3 analysis of 6-plex isobaric tag-labeled whole-cell lysates (WCLs) from WT and *UBR4* KO cells, followed by identification of differentially expressed proteins (DEPs) using MaxQuant software (p-value < 0.05 and >1.5-fold changes as cut-off parameters). Out of 9,695 identified proteins, 654 were significantly increased and 702 were decreased in *UBR4* KO (ΔUBR4) cells. (**B**) Immunoblotting analysis for CDKN1A in multiple clones of ΔUBR4 A549 cells, generated from five different sgRNAs. (**C**) UBR4 was knocked out in the H1975 LUAD cell line (KRAS WT, TP53 mutant), as in A549 cells (KRAS mutant, TP53 WT). (a) Whole-cell lysates (WCLs) from H1975 WT and ΔUBR4 cells were subjected to SDS-PAGE/IB using indicated antibodies. (b) Cell cycle analysis using flow cytometry, quantifying DNA content with DAPI staining. (**D**) As in (C), except the experiments were performed using the H23 (KRAS mutant, TP53 mutant). **(E)** Characterization of a murine LUAD cell line, LLC1, following CRISPR/Cas9-mediated *UBR4* knockout. (a) IB analysis of multiple ΔUBR4 LLC1 clones generated from four different sgRNAs. (b) Cell proliferation rates were assessed in ΔUBR4 cells (clone #1) and control cells (expressing sgCas9 only) by counting trypan blue-stained cells on the indicated time points post-seeding. Shown are representative results from one of three independent biological replicates. ***p < 0.001, two-tailed Student's t-test; ns: not significant. (c) SA-β-gal staining performed on multiple ΔUBR4 LLC1 clones and corresponding Cas9-only control cells. (**F**) GSEA on LUAD samples according to their mutational status in (a) TP53, (b) EGFR, (c) KEAP1, and (d) BRAF. The top hallmark pathways are displayed, with normalized enrichment score (NES) presented on the x-axis. Dot size reflects the gene ratio and dot colors indicate FDR-corrected p-values. Note that, while TP53-mutant cases were divided into quartiles based on UBR4 expression levels, other mutant cases were categorized by the median due to the limited sample size. (**G**) as in (F), except that the GSEA analyses were performed on lung squamous cell carcinoma (LUSC) and small cell lung cancer (SCLC). (**H**) Significantly enriched BPs by ClueGo, including cellular response to chemical stimuli (GO:0070887), mitotic cell cycle process (GO:1903047), chromosome organization (GO:0051276), negative regulation of cell cycle (GO:0045786), and DNA replication (GO:0006260), presented as networks. Each node represents protein sets associated with the same term, with node colors indicating their classification. Node sizes reflect p-values (< 0.05, < 0.005, and < 0.0005 from the smallest to the biggest sizes). (**I**) Functional enrichment analysis (BP) was conducted with DEPs

identified from the TMT-MS analysis using ClueGo. The top 15 GO terms are displayed, with DEPs analyzed by count number (×100). (**J**) Venn diagram illustrating the number of DEGs and DEPs, either upregulated or downregulated, in *UBR4* KO cells. Overlapped DEPs associated with cell cycle and senescence pathways are presented in the boxes. (**K**) Comparison of endogenous proteins in WT and *UBR4* KO cells treated with the proteasome inhibitor MG132 (20 μM for 4 h) before harvesting WCLs. SDS-PAGE followed by immunoblotting (IB) was performed with indicated antibodies, with β-actin/ACTB as a loading control. (**L**) Assessment of CDKN1A/p21 protein degradation in WT and *UBR4* KO cells by monitoring endogenous CDKN1A levels at indicated time points after adding 80 μg/mL cycloheximide (CHX) at time zero. These data complement Figures 1E–1I.

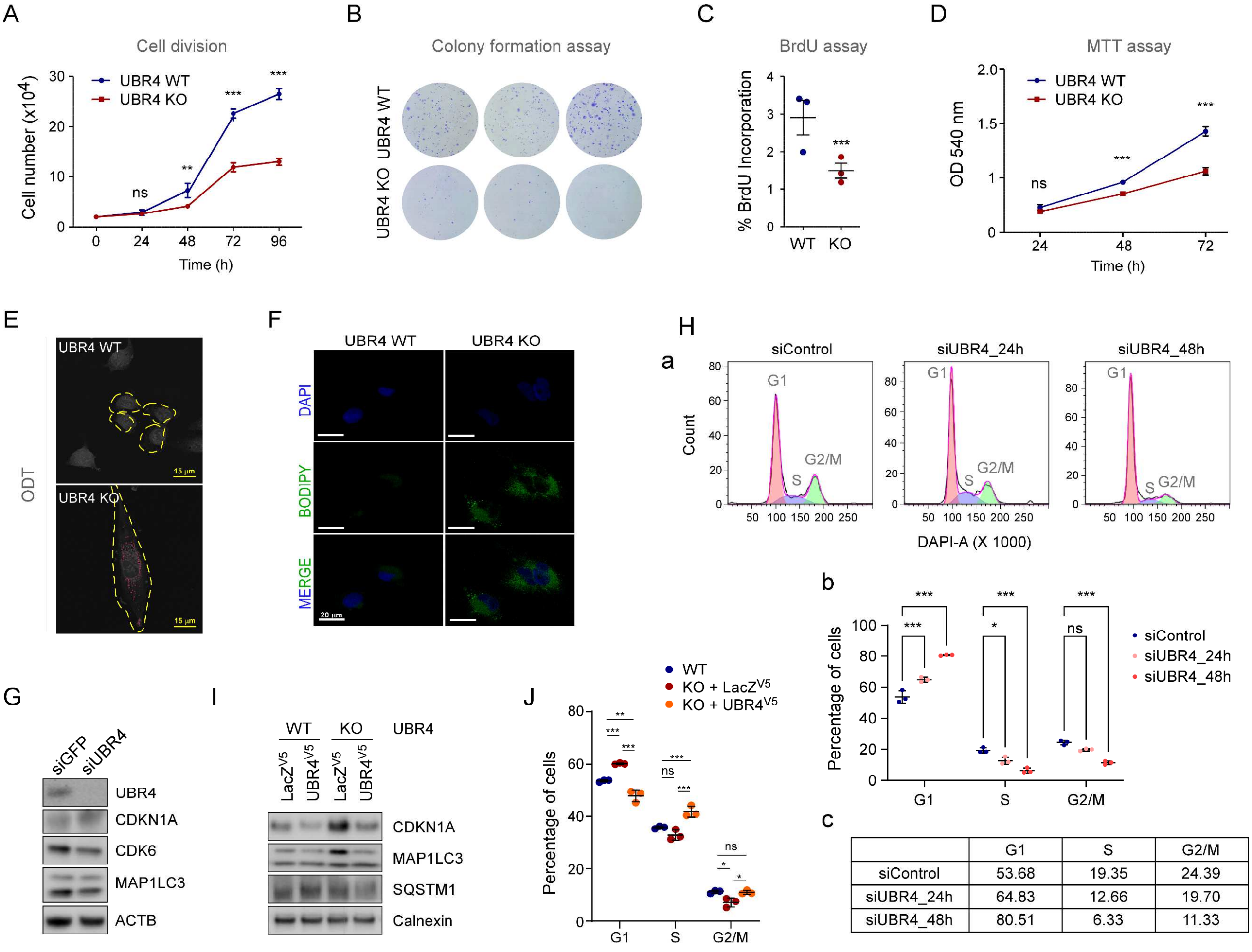

Supplementary Figure 3. Altered cell cycle progression in *UBR4*-deficient cells

(**A**) Growth curves corresponding to WT and UBR4 *KO* cells. Cell proliferation was evaluated in WT and *UBR4* KO cells by counting trypan blue-stained cells on the indicated days post-seeding. Representative data from three independent biological replicates. $^{**}p <$ 0.01, $^{***}p < 0.001$, two-tailed Student's *t*-test; ns: not significant. (**B**) Representative images of colonies formed in A549 WT and *UBR4* KO cells. Cells were cultured for 12 days after low-density seeding (500 cells/well), and colonies were stained with crystal violet. (**C**) ELISA data showing the level of BrdU incorporation. Dot plots depicting the mean absorbance value ± SD at 450 nm ($^{***}p < 0.001$, Student's *t*-test). (**D**) Proliferation rates were assessed using MTT assays at 24, 48, and 72 h on WT and *UBR4* KO cells. Each data point represents the mean ± SD of three independent experiments. (**E**) Representative 3D tomographic images of WT and *UBR4* KO cells based on the refractive index of subcellular organelles. Dotted lines indicate individual plasma membranes. (**F**) Representative images of cytoplasmic neutral lipids in WT and *UBR4* KO cells, stained with BODIPY 493/503 dyes.

Cells were cultured with lipoprotein-depleted serum-containing media for 16 h before BODIPY treatment (1:5,000). (**G**) Endogenous protein levels in siRNA-mediated *UBR4* (si*UBR4*; for 36 h)-silenced HeLa cells were examined via IB using indicated antibodies and compared with control (si*GFP*) cells. (**H**) Time-course analysis of *UBR4* knockdown via siRNA followed by FACS assessment of cell cycle distribution. (a) Flow cytometry histograms depicting DNA content, analyzed using DAPI staining, in HeLa cells treated with siUBR4 for 24 h and 48 h. (b) Quantification of cell cycle phases. *$p < 0.01$, ***$p < 0.001$, compared to untreated cells, based on Bonferroni's multiple comparison ANOVA test. ns, not significant. (c) Summary table presenting the ratio of cells in each cell cycle phase. (**I**) Adding back UBR4 to the *UBR4* KO cells. Cells were transfected with UBR4-expressing plasmids for 36 h, followed by preparation of WCLs, SDS-PAGE separation, and IB with indicated antibodies. (**J**) As in (I), except that flow cytometry-based cell cycle analysis was performed. *$p < 0.05$ and **$p < 0.01$, ***$p < 0.001$ from Bonferroni's multiple comparison ANOVA test. These data complement Figures 2A – 2E.

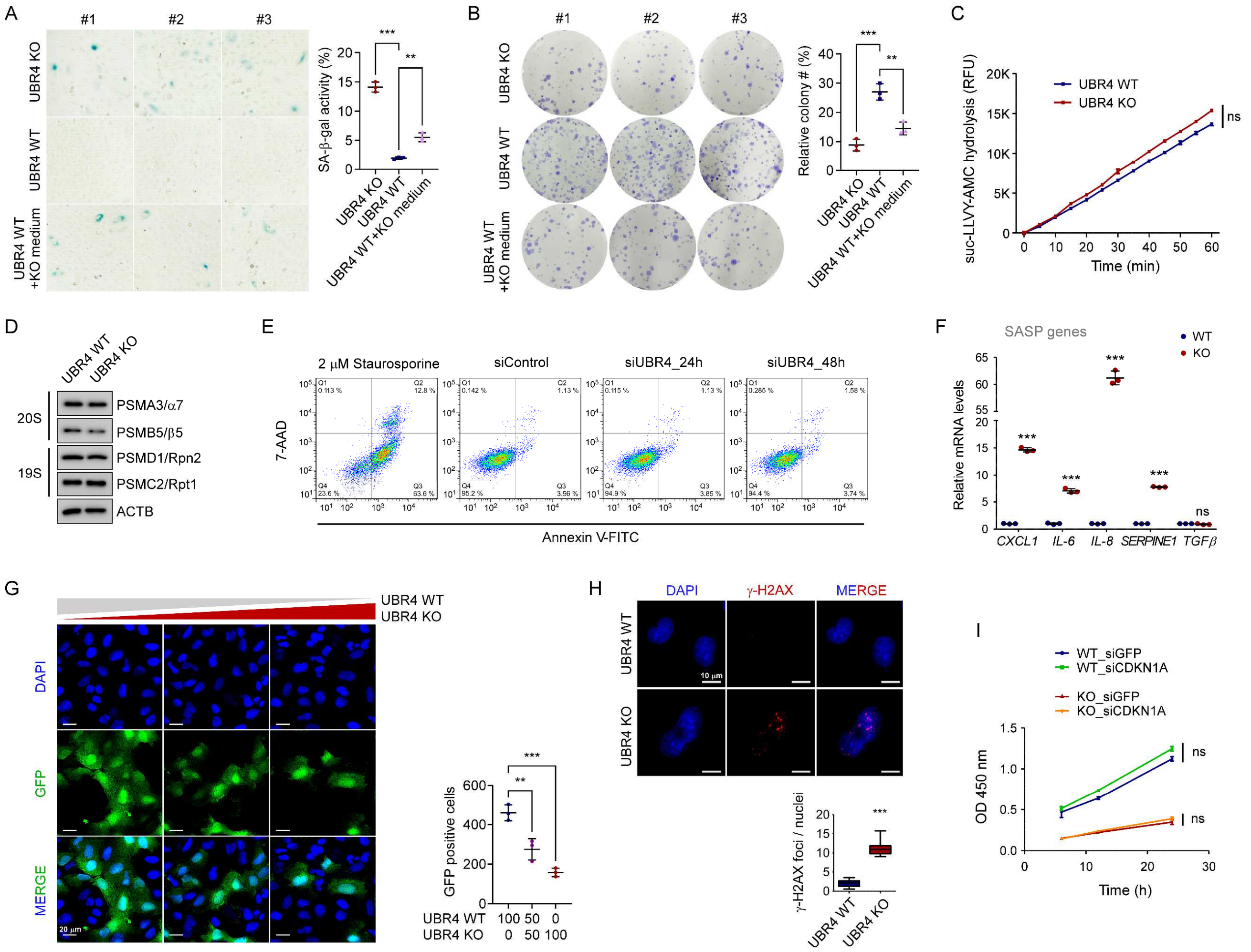

Supplementary Figure 4. Proteasome activity and key pathways in *UBR4*-deficient cells
(**A**) Significantly elevated SA-β-gal positive cell population in WT A549 cells 24 h after treatment with ΔUBR4-conditioned media (CM). Shown are representative images and quantification of SA-β-gal enzymatic staining. $^{**}p < 0.01$ and $^{***}p < 0.001$ from Bonferroni's multiple comparison ANOVA test. (**B**) As in (A), except that a colony formation assay (after 10-day culture with ΔUBR4 CM) was performed. (**C**) Kinetic monitoring of cellular proteasome activity in WT and *UBR4* KO cells via suc-LLVY-AMC hydrolysis using WCLs. A representative result from three independent experiments is shown. RFU, relative fluorescence unit. ns, not significant. (**D**) WCL samples were probed with indicated antibodies targeting the 20S (PSMA3/α7 and PSMB5/β5) or 19S (PSMC2/Rpt1 and PSMD1/Rpn2) complex of 26S proteasome holoenzymes. (**E**) Flow cytometric analysis of apoptotic cells using Annexin V and 7-AAD staining. A549 cells were treated with si*UBR4* for 24 h or 48 h, or with 2 μM staurosporine for 24 h as a positive control. Cells were then stained with Alexa Fluor 488-conjugated Annexin V to detect

phosphatidylserine exposure on the cell membrane, indicative of early apoptosis. (**F**) qRT-PCR was conducted using indicated primers specific for human senescence-associated secretory phenotype (SASP) mRNA. The plotted values are from three independent experiments. *p < 0.05, ** p < 0.01, and ***p < 0.001 from unpaired, two-tailed Student's t-tests. ns: not significant. **(G)** A549 ΔUBR4 significantly inhibited the growth of WT cells, which stably overexpress GFP, in a co-culture assay. Shown are representative fluorescence images (*left*) and quantification of GFP fluorescence intensity (*right*), normalized to WT monoculture (set as 100 %). (**H**) Representative images of γ-H2AX (phosphorylated H2AX at the Ser139) IFS (red) in WT and *UBR4* KO cells with DAPI counter-staining. A quantitative assessment was made by counting all of the foci in 50 randomly chosen cells. Foci per cell values are presented as box (interquartile ranges) and whisker plots (min to max) (***p < 0.001 from two-tailed Student *t*-test). (**I**) WT and *UBR4* KO cells were transfected with either si*GFP* or si*CDKN1A* for 24 h, and their growth was quantified using a CCK-8 assay at indicated time points. These data complement Figures 2F – 2I.

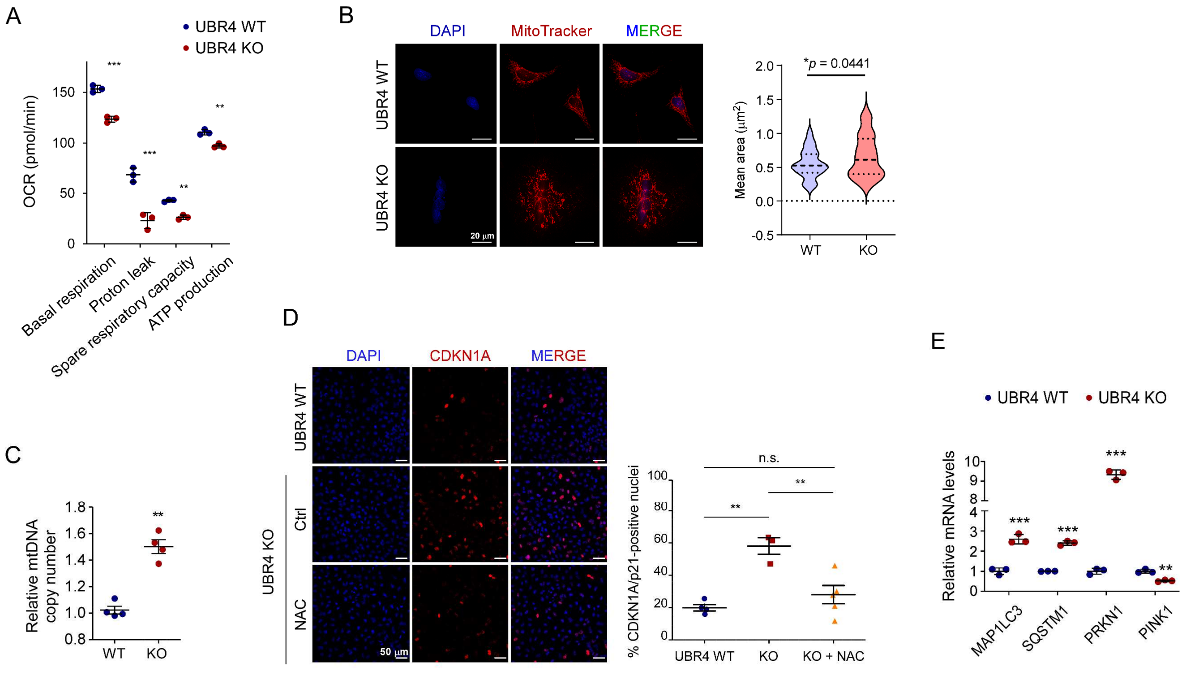

Supplementary Figure 5. Dysfunctional mitochondria and impaired mitophagy in *UBR4*-deficient cells

(**A**) Seahorse analysis of cellular basal respiration, proton leak, maximum respiration, and spare respiratory capacity of WT and ΔUBR4 cells. The relative value of the O_2 consumption rate for each inhibitor treatment is shown as dot plots from three or more independent studies, n = 3 per group. (**B**) (*left*) Mitochondrial morphology was assessed by Mitotracker Red (100 nM for 30 min). Representative images displaying MitoTracker Red positive mitochondria. The scale bars represent 20 μm. (*right*) Violin plot of individual mitochondrial area (Mitotracker Red^{+} area; measured in μm^2), which shows a significant difference between WT and *UBR4* KO cells. p = 0.0441 by t-test with Welch's correction (N = 38 for WT and 82 for KO). (**C**) qRT-PCR analysis of cytosolic mitochondrial DNA (mtDNA). Dot plots with mean ± SD error bars; data were obtained from five independent experiments (n = 5). These data complement Figure 3. (**D**) As in Fig. 3E, except that cells were treated with *N*-acetyl cysteine (NAC). Representative confocal IFS images of WT and *UBR4* KO treated with NAC (100 nM for 24 h), using anti-CDKN1A (green) antibodies with DAPI counter-staining. The percentage of CDKN1A positive cells was quantified and shown as dot plots of three independent experiments with ~500 cells. **$p < 0.01$ and ***$p < 0.001$ from Bonferroni's multiple comparison ANOVA test. (**E**) Comparison of mRNA levels of mitophagy-related genes using qRT-PCR (25 cycles) in biological triplicates. Quantitative normalization was performed using *GAPDH* gene expression.

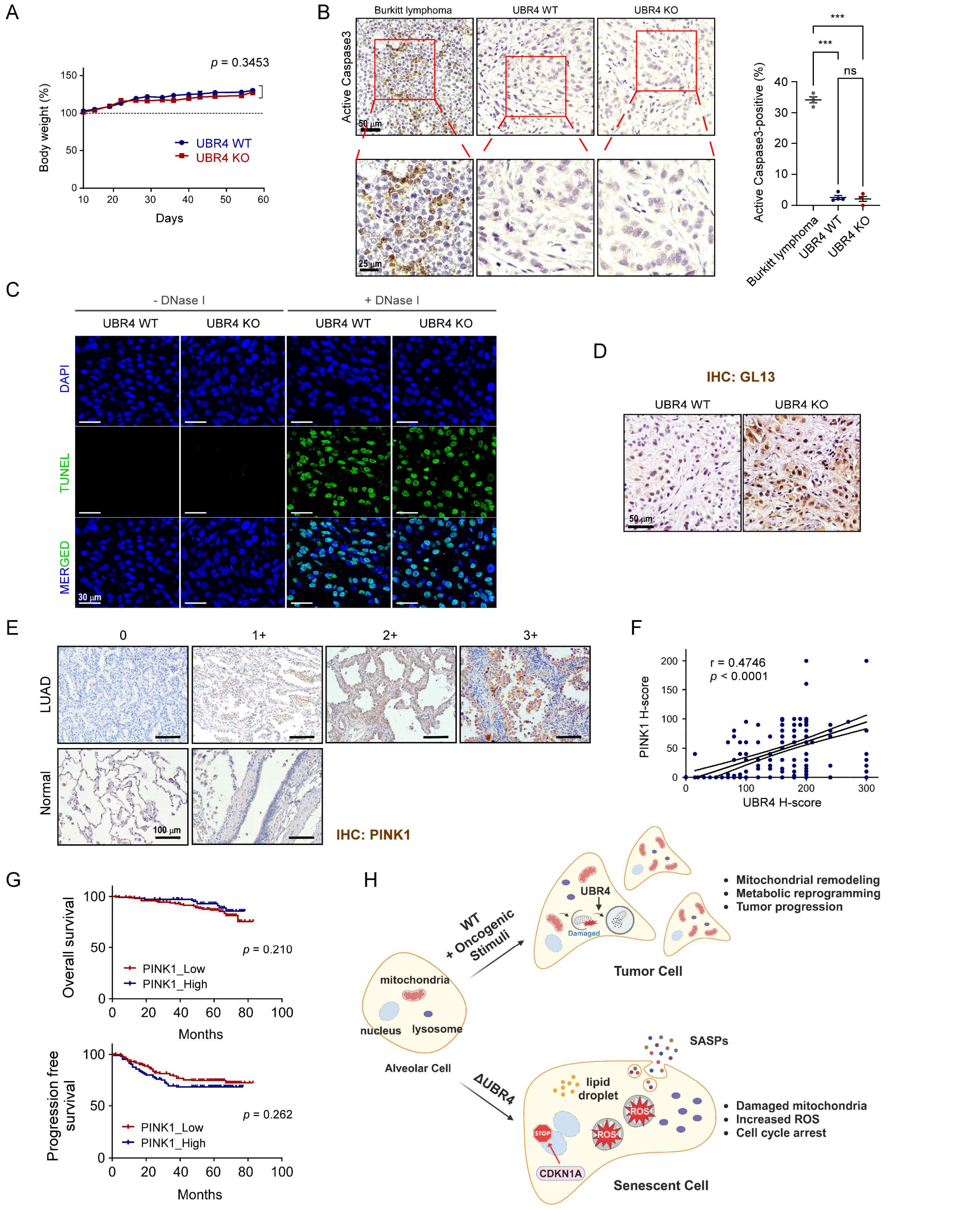

Supplementary Figure 6. Strong correlation of PINK1 and UBR4 in human LUAD tissues

(**A**) Body weight was measured twice a week for 8 weeks from post-implantation. The graph illustrates the body weight changes in mice over the duration of the xenograft experiment. The x-axis

represents the time in days post-implantation while y-axis indicates the percentage change in body weight relative to the initial body weight at the start day. (**B**) Immunohistochemical analysis of cleaved and activated caspase 3 (ASP175 positive) using paraffin sections from *UBR4* WT and KO tumor nodules. A high-grade lymphoma (Burkitt lymphoma) tissues were used as a positive control (32.07 % ± 1.23), showing mostly cytoplasmic active-caspase 3 signals with apoptotic cell morphologies. Representative images (*left*) and quantitative analysis (*right)* for each group are displayed. ns, not significant (N = 3, Student's *t*-test). **(C)** TUNEL assay of UBR4 WT and KO tumor tissues with or without DNase I treatment. Representative images of WT and KO tissues are shown under DNase I as positive control. (**D**) As in (B), except GL13 staining was performed to detect cellular senescence. **(E)** Representative images showing the expression of PINK1 protein in LUAD and normal lung tissues. The panels display different staining intensities, graded as 0, 1+, 2+ and 3+, indicating negative to strong positive staining, respectively. The scale bar represents 100 μm. **(F)** The scatter plot depicts the correlation between the expression level of UBR4 and PINK1 proteins in LUAD tissues (N = 250; r = 0.4746, p < 0.0001 from Spearman correlation analysis). Solid and dashed lines depict regression lines and 95% confidence intervals, respectively. **(G)** Kaplan-Meier plots showing overall survival and progression-free survival of LUAD patients based on PINK1 expression. Patients were divided into two groups: low expression (≤ median H-score; 60) and high expression (> median H-score) of PINK1. Log-rank test p-values are indicated for each comparison. (**H**) Schematic representation outlining the tumorigenic function of UBR4. UBR4 promotes cellular mitochondrial and metabolic remodeling via mitophagy, which is instrumental in tumorigenesis under chronic stress conditions or oncogenic stimuli. In contrast, UBR4-deficient cells exhibit aberrant mitochondrial dynamics and elevated ROS levels, ultimately resulting in cell cycle arrest potentially through the TP53-CDKN1A pathway. Therefore, the UBR4-mitophagy axis critically coordinates the reciprocal pathways determining tumorigenesis and senescence.

SUPPLEMENTARY TABLES

Supplementary Table 1. Clinical and pathological features of patients with LUAD (n=250)

Characteristics		N (% or range)
Age (years)	Median (range)	63.8 (36-84)
Gender	Male	110 (44.0)
	Female	140 (56.0)
Smoking	Never	157 (62.8)
	Ever	93 (37.2)
Tumor size (cm)	Median (range)	2.7 (1.0-11.3)
Lymph node metastasis	Absent	193 (77.2)
	Present	45 (18.0)
Stage	I	182 (72.8)
	II	22 (8.8)
	III	34 (13.6)
	IV	12 (4.8)
Lymphatic invasion	Absent	189 (72.4)
	Present	61 (27.6)
Venous invasion	Absent	223 (89.2)
	Present	27 (10.8)
Perineural invasion	Absent	239 (95.6)
	Present	11 (4.4)

Supplementary Table 2. Correlation between clinicopathological features and expression of PINK1 and UBR4 in LUAD

	PINK (H-score 60)			UBR4 (H-score 150)		
	**Low (N=145)**	**High (N=105)**	***p***	**Low (N=78)**	**High (N=172)**	***p***
**Gender**						
Male	62 (56.4)	48 (43.6)	0.642	29 (26.4)	81 (73.6)	0.143
Female	83 (59.3)	57 (40.7)		49 (35.0)	91 (65.0)	
**Age**						
<60	46 (61.3)	29 (38.7)	0.485	27 (36.0)	48 (64.0)	0.284
≥ 60	99 (56.6)	76 (43.4)		51 (29.1)	124 (70.9)	
**Smoking**						
Never	94 (59.9)	63 (40.1)	0.436	52 (33.1)	105 (66.9)	0.394
Ever	51 (54.8)	42 (45.2)		26 (28.0)	67 (72.0)	
**Tumor size**						
<3cm	101 (60.5)	66 (39.5)	0.260	56 (33.5)	111 (66.5)	0.259
≥3cm	44 (53.0)	39 (47.0)		22 (26.5)	61 (73.5)	
**Lymph node metastasis**						
N0	112 (58.0)	81 (42.0)	0.617	65 (33.7)	128 (66.3)	0.157
N1	24 (53.3)	21 (46.7)		10 (22.2)	35 (77.8)	
**Lymphatic invasion**						
No	104 (57.5)	77 (42.5)	0.779	58 (32.0)	123 (68.0)	0.641
Yes	41 (59.4)	28 (40.6)		20 (29.0)	49 (71.0)	
**Venous invasion**						
No	128 (57.4)	95 (42.6)	0.580	72 (32.3)	151 (67.7)	0.286
Yes	17 (63.0)	10 (37.0)		6 (22.2)	21 (77.8)	
**Perineural invasion**						
No	137 (57.3)	102 (42.7)	0.366	76 (31.8)	163 (68.2)	0.510
Yes	8 (72.7)	3 (27.3)		2 (18.2)	9 (81.8)	

Supplementary Methods

Antibodies

Antibodies, catalog numbers, and dilution factors used in this study are as follows: anti-ACTB (Millipore Sigma, A1978, 1:10,000), anti-GAPDH (Santa Cruz Biotechnology [SCBT], sc-32233, 1:5,000), anti-UBR4 (Abcam, ab-86738, 1:1,000 for IB, 1/250 for IHC), anti-CDKN1A (Cell Signaling Technology [CST], 2947, 1:2,000 for IB, 1:500 for IFS, 1:200 for IHC), anti-CDK2 (CST, 2546, 1:1,000), anti-CDK6 (CST, 3136T, 1:1,000), anti-CDKN2A (SCBT, sc-1661, 1:2,000), anti-TIMM23 (Proteintech, 11123-1-AP, 1:4,000 for IB, 1:100 for IFS), anti-PPARGC1A (Abclonal, A12348, 1:2,000), anti-TP53 (Abcam, ab126, 1:2,000), anti-phospho-TP53 (Thermo Fisher Scientific [TFS], 700439, 1:1,000), anti-γ-H2AX (Abclonal, AP0099, 1:1,000 for IB, 1:100 for IFS), anti-α-tubulin (SCBT, sc-5286, 1:2,000), anti-Ub (SCBT, sc-8017, 1:3,000), anti-MAPLC3 (Millipore Sigma, L7543, 1:2,000), anti-V5 (TFS, R960-25, 1:500), anti-SQSTM1 (Abcam, ab56416, 1:2,000), anti-calnexin (Bethyl Laboratories, A303-694A, 1:5,000), anti-PSMD1 (SCBT, sc-514809, 1:2,000), anti-PSMA3 (Enzo Life Sciences, BML-PW8110, 1:2,000), anti-PSMB5 (TFS, PA1-977, 1:2,000), anti-PSMC2 (SCBT, sc-166972, 1:2,000), anti-OPA1 (Proteintech, 27733-1-AP, 1:2,000), anti-PRKN (CST, 4211, 1:1,000 for IB, 1:40 for IHC), anti-PINK1 (Proteintech, 23274-1-AP, 1:1,000 for IB, 1:250 for IHC), anti-CYC1 (Abclonal, A13430, 1:1,000), anti-TUBB (SCBT, sc-55529, 1:1,000), anti-DNA (Progen, 690014, 1:500), anti-Ki67 (Abclonal, A20018, 1:500), anti-phospho-Ub (Ser65) (Millipore, ABS1513-I, 1:2,000), and anti-active caspase 3 (ASP175; CST, 9661, 1:50). The secondary antibodies for IB (horseradish peroxidase [HRP]–conjugated anti-mouse IgG and anti-rabbit IgG antibodies) and for IFS (goat anti-rabbit Alexa Fluor 488/594 IgG and goat anti-mouse Alexa Fluor 488/594 IgG) were acquired from Millipore Sigma and TFS, respectively.

Analysis of GSEA data

RNAseq data for the TCGA LUAD subsets were sourced from cBioportal (https://www.cbioportal.org/), comprising gene expression profiles for 510 tumor samples and 59 normal samples. The cohort of tumor samples was categorized into quantiles based on UBR4 gene expression levels, resulting in UBR4_high (N = 127) and UBR4_low (N = 127) groups. Hallmark gene sets were curated from MSigDB (https://www.gsea-msigdb.org/gsea/msigdb/), and GSEA was performed using GSEA software (ver. 4.1.0). p-Values were determined by permuting the data 1,000 times to identify enriched gene sets. The GSEA software generated enrichment score, normalized enrichment score (NES), nominal p-value, and false discovery rate (FDR). Co-expression data for

genes associated with the cell cycle and mitochondria were extracted from TCGA via the cBioportal database. Patients were stratified into UBR4_high and UBR4_low groups, and differential expression analysis was conducted to identify significantly upregulated or downregulated DEGs within each group. After identifying DEGs, GO analysis was carried out using the DAVID bioinformatics resources, focusing on the terms of ‘biological processes’ and selecting the top seven processes based on fold enrichment scores. Kaplan–Meier survival curves associated with UBR4 expression were constructed for LUAD patients using GraphPad Prism (ver. 9.3.1).

Cell culture, transfection, and reagents

Human LUAD A549, H1975, H23 cells were cultured in RPMI 1640 (Welgene, LM011); HeLa and LLC1 cells were cultured in DMEM (Welgene, LM001), supplemented with heat-inactivated 10% FBS (TFS, A3382001), 2 mM L-glutamine (Welgene, LS002), and 100 U/mL penicillin/streptomycin in a humidified atmosphere with 5% CO2 at 37°C. Routine mycoplasma contamination testing was performed using DAPI staining kit (Abcam, ab104139). For transient overexpression, cells were transfected with 1.5 – 3 μg of total plasmid DNA in a 12-well or 6-well culture plate using Lipofectamine 3000 transfection reagent (TFS, L3000075), following the manufacturer's instructions. Unless otherwise stated, all reagents were applied to the cells as follows: CM-H2DCFDA (10 μM; TFS, C6827), N-acetyl-L-cysteine (20 μM; Bio Basic, AB6697), mito-TEMPO (100 nM; Sigma, SML0737), FCCP (10 μM; Apexbio, B5004), MG132 (20 μM; AG Scientific, M-1157), staurosporine (2 μM; Apexbio, A8192) and cycloheximide (80 μg/mL; Cayman Chemical, 14126).

Sample preparation for proteomics analysis.

Cell pellets were lysed in protein extraction buffer containing 4% SDS, 2 mM tris-(2-carboxyethyl)-phosphine (TCEP), and 0.1 M Tris-HCl (pH 7.5). Whole-cell lysates (WCLs) were sonicated and heated for 30 min at 95°C. Protein concentration was determined using a reducing agent-compatible BCA assay. Subsequently, each sample containing 200 μg of total protein was precipitated with 5 volumes of cold acetone. Samples were then resuspended in denaturation buffer (2% SDS, 10 mM TCEP, 50 mM chloroacetamide, and 0.1 M Tris-HCl [pH 8.5]) and heated for an additional 15 min at 95°C. Protein digestion was performed via multistep digestion utilizing filter-aided sample preparation, as previously described [1]. Digested samples were loaded onto a 30 K spin filter, with subsequent buffer exchanges using UA buffer (8 M urea in 0.1 M Tris-HCl [pH 8.5]) and 50 mM triethylammonium bicarbonate (TEAB) solution. The first protein digestion was performed overnight

at 37°C using the Trypsin/LysC mix (Promega; protein-to-protease ratio = 100:1), followed by peptide collection. The remaining proteins underwent a second digestion at 37°C for 3 h using trypsin (protein-to-protease ratio = 1000:1), with subsequent peptide collection and an additional elution step with 100 µL of 50 mM TEAB.

Tandem mass tag (TMT) labeling and desalting

TMT labeling was conducted per the manufacturer's protocol, with minor adjustments. The TMT reagent (0.8 mg) was dissolved in 100% acetonitrile. After spiking with peptides derived from ovalbumin as an internal standard, 25 µL of the reagent was added to 50 µg of peptide samples and acetonitrile to give a final concentration of 30% (v/v). WT samples were labeled with TMT-126, TMT-127, and TMT-128, whereas ΔUBR4 samples were labeled with TMT-129, TMT-130, and TMT-131. After incubation at RT for 1 h, the reaction was quenched with 5% hydroxylamine. TMT-labeled samples were pooled at a 1:1:1:1:1:1 ratio, vacuum-centrifuged, and subjected to OASIS HLB solid-phase extraction.

Offline high pH reversed-peptide fractionation

TMT-labeled peptide mixtures were fractionated using high-pH peptide reverse-phase fractionation (Agilent 1290 Bio-Inert HPLC with an analytical column [4.6 µm × 250 mm, 5 µm]). Solvent A consisted of 15 mM ammonium hydroxide in water, and solvent B consisted of 15 mM ammonium hydroxide in 90% acetonitrile. Peptides were separated with a 5 – 35% acetonitrile gradient at 0.2 mL/min flow rate. In total, 96 fractions were pooled into 24 samples, which were lyophilized and stored at −80°C until MS/MS analysis.

Mass spectrometry (MS) and data processing

The 24 peptide fractions underwent analysis using an LC-MS system (Orbitrap Exploris 480, TFS) equipped with an Ultimate 3000 RSLC system (Dionex). EASY-Spray LC columns (TFS) served as the electrospray source, with the column heater temperature set to 60°C. Peptides were separated using a 2-column system comprising a trap column (3 mm diameter and 1 cm length) and an analytic column (75 µm diameter and 50 cm length). Separation occurred over a 180-min gradient from 7 – 32% solvent B at a flow rate of 300 nL/min. Survey MS scans were conducted in the 350 – 1650 m/z range with a resolution of 120,000 at m/z 200. Data acquisition employed a data-dependent mode using a top 20 method to select the 20 most abundant precursor ions with an isolation width of 0.7 m/z. High-energy collisional dissociation scans utilized a normalized collision energy of 32 and a

resolution of 30,000 at 200 m/z. Maximum ion injection times for the survey and MS/MS scans were 25 and 85 ms, respectively. Raw MS/MS files were processed with Proteome Discoverer ver. 2.5 (TFS), using the SEQUEST HT algorithms against the UniProt database. Database search parameters included 1) complete enzyme digest using trypsin with up to two missed cleavages allowed, 2) a precursor ion mass tolerance of 20 ppm, 3) a fragment ion mass tolerance of 0.02 Da, 4) dynamic modifications of 15.995 Da for methionine oxidation and 42.011 Da for protein N-terminal acetylation, 5) static modifications of 57.021 Da for carbamidomethylation on cysteine residues, and 6) 229.153 Da for TMT 6-plex on any N-terminus. The MS1 co-isolation threshold was set to 50%, and six reporter ion intensities for TMT were corrected for isotopic impurities. Percolator analysis confirmed peptide spectral matches and peptides based on a 1% FDR. Confidence criteria were set to a 1% FDR at the protein level. The MS-based proteomics data, encompassing all identified peptides and protein lists, were deposited in the ProteomeXchange Consortium (http://proteomecentral.proteomexchange.org) via the PRIDE partner repository (Identifier #PXD051417) [3].

Analysis of cell cycle distribution

For flow cytometry analysis, cells were trypsinized, collected in phosphate buffered saline (PBS), washed with ice-cold PBS, and fixed in 70% ice-cold ethanol overnight. Fixed cells were then centrifuged at 780 × g for 5 min, resuspended in PBS, and incubated with DAPI/Triton X-100 staining solution at room temperature (RT) for 30 min in the dark [2]. The cells were then analyzed using LSRFortessa (BD Bioscience) with UV light excitation at 340 – 380 nm, and data analysis was performed using Flowjo software (ver. 10.9.0). The DNA content histograms were plotted, and the percentage of cells in each phase (G0/G1, S, and G2/M) was determined. For the EdU staining assay, cells were incubated with EdU for 2 h to label actively synthesizing DNA, fixed, permeabilized, and treated using the Click-iT kit (TFS, C10269). The quantification of EdU positive cells after fluorescence microscopy imaging was represented as mean ± SEM. Nuclei were counterstained with DAPI. BrdU incorporation was analyzed according to the manufacturer's instructions (Roche, 11647229001). Briefly, the cells were incubated with 10 μM BrdU for 24 h at 37°C in a 5% CO2 incubator, washed three times, and fixed with FixDenat solution. The cells were then incubated with a peroxidase-conjugated anti-BrdU antibody for 90 min at RT. Following washes, the substrate for conjugated peroxidase was added. The reaction product was quantified by measuring absorbance at 450 nm using a microplate reader (Tecan Infinity 200 PRO).

Electron microscopy (EM) imaging

Samples for EM analysis were prepared following a standard fixation and embedding protocol as previously described [54]. Briefly, after fixation with 2.5% glutaraldehyde in 0.1 M phosphate buffer at pH 7.4, WT and ΔUBR4 cells were washed in PBS for 10 min and post-fixed in 1% osmium tetroxide at RT for 2 h. Subsequently, samples were dehydrated in a graded series of ethanol solutions (50%, 60%, 70%, 80%, 90%, 95%, and 100% ethanol), followed by two changes in 100% acetone. Samples were then embedded in EmBed 812 resin, and ultrathin slices were prepared using PowerTome (RMC Boeckeler) and collected on a copper grid. These sections were stained with 4% uranyl acetate and 4% lead citrate before EM images were captured at magnifications ranging from 8,000× to 25,000×, using a JEOL JEM-1400 transmission electron microscope at an accelerating voltage of 80 kV. For image analysis, mitochondria were classified based on specific morphological criteria: mitochondria with swollen or disrupted cristae or diameters <500 nm were classified as abnormal. Quantification was performed by calculating the abnormal to normal mitochondria ratio in each sample, providing a measure of mitochondrial integrity and health under various experimental conditions.

Quantitative RT-PCR (qRT-PCR) and RNA sequencing (RNAseq)

Total RNA extraction from the cultured cells was performed using TRIzol reagents and additional purification using the AccuPrep Universal RNA Extraction Kit (Bioneer, K-3140, Bioneer) with on-column DNase I treatment. For qRT-PCR, cDNA samples were prepared by RT-PCR (Accupower RT-pre-mix, Bioneer, K-2041) using 2 μg of total RNA. qRT-PCR reactions were conducted with 1/20 diluted cDNA, SYBR (TOPreal PreMIX, Enzynomics, RT500) as the reporter dye, and 10 pmol of primers to detect mRNA expression of gene-of-interests. Each mRNA level was normalized to that of *GAPDH*. Unpaired Student's *t*-tests evaluated results, with *p*-values < 0.05 considered statistically significant. The target gene-specific primer sequences were as follows: for *GAPDH*, forward 5'-AGGGCCCTGACAACTCTTTT-3' and reverse 5'- AGGGGTCTACATGGCAACTG-3'; for *CDKN1A*, forward 5'-TGTCCGTCAGAACCCATGC-3' and reverse 5'-AAAGTCGAAGTTCCATCGCTC-3'; for *CDKN2A*, forward 5'-CGCAGGTTCTTGGTCACTGT-3' and reverse 5'- TGTTCACGAAAGCCAGAGCG-3'; for *TP53*, forward 5'-CAGCACATGACGGAGGTTGT-3' and reverse 5'-TCATCCAAATACTCCACACGC-3'; for *CXCL1,* forward 5'-AGGGAATTCACCCCAAGAAC-3' and reverse 5'-ACTATGGGGGATGCAGGATT-3'; for *IL-6*, forward 5'-ACTCACCTCTTCAGAACGAATTG-3' and reverse 5'-CCATCTTTGGAAGGTTCAGGTTG-3', for *IL-8*, forward 5'-ACTGAGAGTGATTGA GAGTGGAC-3', reverse 5'-AACCCTCTGCACCCAGTTTTC-3'; for

SERPINE1, forward 5'-ACCGCAACGTGGTTTTCTCA-3' and reverse 5'-TTGAATCCCATAGCTGCTTGAAT-3'; for *TGFβ*, forward 5'-GGC CAG ATC CTG TCC AAG C-3' and reverse 5'-GTG GGT TTC CAC CAT TAG CAC-3'; for *MAP1LC3*, forward 5'-AAGGCGCTTACAGCTCAATG-3' and reverse 5'-CTGGGAGGCATAGACCATGT-3'; for *SQSTM1*, forward 5'-GACTACGACTTGTGTAGCGTC-3' and reverse 5'-AGTGTCCGTGTTTCACCTTCC-3'; for *PRKN*, forward 5'-GGTTTGCCTTCTGCCGGGAATG-3' and reverse 5'-CTTTCATCGACTCTGTAGGCCTG-3'; for *PINK1*, forward 5'-GGGGAGTATGGAGCAGTCAC-3' and reverse 5'-CATCAGGGTAGTCGACCAGG-3'; for *ATP8A*, forward 5'-ATGGCCCACCATAATTACCC-3' and reverse 5'-TTTTATGGGCTTTGGTGAGG-3'. Unpaired Student's *t*-tests were performed to evaluate the results, where *p*-values of less than 0.05 were considered statistically significant. For RNAseq, the cDNA library was constructed using a QuantSeq 3′-mRNA-Seq Library Prep Kit (Lexogen) and sequenced on the Hiseq platform (Illumina). Normalization and quantitative analysis were performed using the DESeq2 software package (https://www.bioconductor.org/packages/release/bioc/html/DESeq2.html) [4]. DEGs were identified based on a threshold of at least a 1.6-fold change, with a significance level of $p < 0.05$.

RNA interference

The siRNAs targeting CDKN1A (sc-29427) and control siRNA targeting GFP (sc-45924) were obtained from SCBT. The siRNA targeting UBR4 (sense: 5'-CAACAUCUGCCCUUCAAAUUU-3', antisense: 5'-AUUUGAAGGGCAGAUGUUGUU-3'; designated as si*UBR4*) was manufactured by Genolution. Upon reaching 70 – 80% confluency, siRNAs (final 30 nM) were transfected using RNAiMAX (TFS, #13778150) dissolved in Opti-MEM (TFS, #31985070). After 24 h post-transfection, cells were trypsinized, suspended in the transfection mixture, re-plated, and re-transfected with the siRNA duplexes for an additional 48 h, which provided more effective knockdown [5].

Comprehensive analysis of RNAseq and TMT-MS

For generating heatmaps, the raw protein measurements were first averaged across six data sets, encompassing three biological replicates for both WT and ΔUBR4 cell samples. Then, the analysis focused on DEPs and DEGs relevant to cell cycle and senescence, sourced from the MitoCarta 3.0 database. Venn diagrams were constructed using Venny 2.1.0 (http://bioinfogp.cnb.csic.es/tools/venny/index.html). Z-scores were calculated to evaluate the deviation of each data point from the mean, employing normalization to account for protein-to-

protein variation. Volcano plots were created to visualize RNAseq analysis, where the x-axis represented the log2 fold change and the y-axis depicted the negative logarithm of the *p* value, using GraphPad Prism. For cytoscape analysis, TMT-MS data were analyzed using ClueGO applications (ver. 2.5.10) within the Cytoscape environment (ver. 3.10.1). DEPs were utilized to construct biological networks focusing on biological processes, with a kappa score threshold of 0.55, a GO level set between 2 and 5, and a p value < 0.05. Parameters for GO Term/Pathway Selection included a minimum of 8 genes and a kappa score of 0.55 for GO term grouping, with 25% thresholds for merging groups. Statistical significance was determined at $p = 0.05$, with the Benjamin-Hochberg adjustment applied. TCGA RNAseq data for genes coexpressed with *UBR4* were analyzed using ClueGO, with a kappa score of 0.7 and GO levels adjusted to level 4, aiming to elucidate networking and associated biological processes.

Optical diffraction tomography

To obtain refractive index-based optical diffraction tomograms, cells were plated on 50 mm imaging dishes with a #1.5H glass coverslip bottom (TomoDish, Tomocube) and stabilized in an incubator at 37°C for 24 h to maintain a confluence of approximately 60%. The phase shifts caused by cellular components in the WT and ΔUBR4 cells were captured using HT-2H (Tomocube). By a phase retrieval algorithm, the amplitude and phase images of the cells were retrieved from the obtained holograms. The whole cell volume and lipid droplet volume were quantitatively assessed using TomoStudio software (ver. 2.7.30). Statistical analysis was performed to evaluate the significance of observed differences. The mean values and standard deviations were calculated for each parameter, and the Student’s t-test was applied to assess the statistical significance between the two groups.

SDS-PAGE and immunoblotting (IB)

Cultured cells were washed twice with PBS and lysed in RIPA buffer (150 mM NaCl, 0.5% sodium deoxycholate, 0.1% SDS, 1% NP-40, 50 mM Tris-HCl [pH 8.0]) supplemented with a protease inhibitor cocktail and phosphatase inhibitor sodium as supernatants, which were centrifuged at 14,000 × g for 30 min at 4°C to collect WCLs. For SDS-PAGE, WCLs were mixed with 5× SDS sample buffer (final 10% glycerol, 2% SDS, 0.05% bromophenol blue, 50 mM Tris-HCl [pH 6.8], and 5% 2-mercaptoethanol) and denatured at 85°C for 10 min. Proteins were separated by SDS-PAGE and transferred to a 0.45 μm polyvinylidene difluoride membrane. For UBR4, gel separation was performed with NuPAGE BisTris 4 – 12% precast gels with MES Running buffer (TFS, NP0323BOX) as previously described [5]. After blocking with 2% bovine serum albumin (BSA) in

TBST (20 mM Tris-HCl [pH 7.5], 150 mM NaCl, and 0.1% (w/v) Tween 20) solution, membranes were incubated overnight with primary antibodies. Following three washes with TBST, membranes were incubated with HRP-conjugated anti-rabbit IgG or anti-mouse IgG antibody for 60 min at RT. After antibody incubation, bands were visualized via chemiluminescence, imaged, and quantified.

Immunofluorescence microscopy

For IFS, cells cultured on cover glass were fixed with 4% paraformaldehyde (PFA) in PBS for 15 min, followed by permeabilization with 0.5% (v/v) Triton X-100 in PBS. After blocking with 2% BSA in PBS for 30 min, cells were incubated overnight at 4°C with primary antibodies. After further washing with PBS, cells were incubated with Alex Fluor 488- or Alex Fluor 594-conjugated secondary antibodies (1:1,000) for 40 min. Cells were mounted using a DAPI-containing mounting solution (Abcam, ab104139) and examined under a fluorescence microscope. To detect lipid droplets, we labeled them with BODIPY 493/503 (1:5,000 from a 1 mg/mL stock solution in DMSO; TFS, D3922) for 15 min after cell fixation.

Cell proliferation assay

WT and ΔUBR4 cells were seeded with 2 x 10^4 cells in a well of 24-well plates and cultured for 24, 48, 72, and 96 h. After harvesting the cells using trypsin-EDTA they were resuspended with 0.4% trypan blue (1:1 volume ratio) by gentle pipetting, and then 20 μL of the mixture was loaded into each chamber of the hemocytometer. Counting was performed in triplicate by one operator under a microscope with a 40× objective. For the colony forming assay, 500 cells were seeded in a 60 mm dish at 37°C with 5% CO_2 for 48 h. After 12 days, colonies were washed with cold DPBS twice, fixed with 2 mL of cold methanol for 30 min, and stained with 1% crystal violet for 5 min at RT. Stained colonies with >50 cells were counted. Plating efficiency was calculated as (number of colonies /500 cells) x 100 (%). Cell viability was also assessed with a modified 3-(4,5-dimethylthiazol-2-yl)-2,5-diphenyltetrazolium bromide (MTT) assay in a triplicated manner [6]. Briefly, cells were seeded in 96-well plates at 2 x 10^3 cells per well, cultured for 24, 48, or 72 h, and incubated with 100 mL of MTT (0.5 mg/mL in RPMI) for 2.5 h at 37°C. Absorbance of blue formazan crystals solubilized by 200 μL of DMSO was measured at 570 nm for test and 630 nm for reference wavelength. ΔUBR4-conditioned media (CM) was collected from A549 ΔUBR4 cell cultures. Collected CM was centrifuged at 780 x g for 3 min, filtered through a 0.45 μm filter to remove cell debris, and stored at -80°C for future experiments. For SA-β-gal staining and colony

formation assay, the culture media of WT A549 cells at 35% confluence was replaced with ΔUBR4 CM.

Immunohistochemical (IHC) analysis

Xenograft tumor tissues of WT and ΔUBR4 cells were assessed by immunohistochemistry. Mouse xenograft tumors were harvested, fixed with 4% PFA in PBS at 4°C, dehydrated, and embedded in paraffin. Paraffin blocks were sectioned at 4 μm thickness and mounted on silane-coated slides. After deparaffinization and rehydration, antigen retrieval was performed using citrate buffer (pH 6.0 for MKI67 and CDKN1A; pH 9.0 for UBR4, PINK1, and PRKN) in a microwave for 20 min, followed by blocking of non-specific binding with Animal-Free Blocker (Vector Laboratories, SP-5035-10) for 30 min at 4°C. To detect active caspase 3, anti-cleaved caspase 3 (ASP175; Cell Signaling) was used. Primary antibody incubation was carried out overnight at 4°C, followed by incubation with secondary antibody (a biotinylated goat anti-mouse and rabbit antibody [1:200; Vector laboratory, BA-9200 and BA-1000, respectively]) for an additional 30 min. The slides were visualized with an ABC-HRP kit (Vector Laboratories, PK-4000) with 3,3′-diaminobenzidine (Vector Laboratories, SK-4105) as the chromogen and the images were captured using an Aperio AT2 slide scanner (Leica Biosystems). Quantification of staining was performed using ImageJ software.

Co-culture assay

To assess paracrine effects, A549-ΔUBR4 cells were co-cultured with WT cells stably expressing EGFP (A549-EGFP) in 24-well plates at a density of 1×10^4 cells/well. After allowing the cells to adhere, co-cultures were maintained under standard conditions for 48 h. Fluorescence microscopy images were acquired, and GFP signals were analyzed using ImageJ for quantitative visualization and statistical analysis.

Apoptosis analysis

For *in vitro* apoptosis assay, cells were seeded in 6 well-plates and either treated with 2 μM staurosporine for 24 h (positive control) or transfected with siRNAs using Lipofectamine RNAiMAX. After the indicated incubation period, both floating and adherent cells were collected, washed with ice-cold PBS, and stained with Annexin V-FITC (TFS, BMS500FI-100) and 7-AAD (BioLegend, 420404), following the manufacturer’s protocols. Samples were analyzed within 1 h using a BD LSRFortessa flow cytometer, and data were processed with FlowJo software (ver. 10.9.0). Apoptotic cells were defined as Annexin V-positive/7-AAD-

negative (early apoptosis) or Annexin V- and 7-AAD-double positive (late apoptosis). For *in vivo* detection of apoptosis, active caspase-3 was detected by IHC with Burkitt lymphoma tissues as a positive control to validate staining specificity. Terminal deoxynucleotidyl transferase dUTP nick end labeling (TUNEL) staining was performed on paraffin-embedded sections using the TUNEL Andy Fluor 488 Apoptosis Detection Kit (ABP Biosciences, A050), according to the manufacturer's instructions. Nuclei were counterstained with DAPI and visualized using a confocal microscope.

Assessment of mitophagy

For analysis of mitophagy, cells were transfected with GFP-MAP1LC3 plasmids for 30 h and incubated with MitoTracker Red CMXRos dye for 30 min. Subsequently, the cells were fixed and imaged using confocal microscopy. Colocalization profiles were normalized to the maximum intensity of both mitochondria and MAP1LC3. For colocalization assay of lysosomes and mitochondria, cultured cells were incubated with 100 nM LysoTracker Red DND-99 for 1 h, subsequently fixed and permeabilized. After blocking, cells were incubated with anti-TIMM23 antibodies. For the mito-Keima assay, the cells were first transduced with Lenti-mito-Keima for 48 h and selected with 8 μg/mL of blasticidin to establish a stable cell line. Following treatment with 10 μM FCCP for 6 h in WT and ΔUBR4, quantitative colocalization analysis was performed using Zeiss LSM980 Airyscan2 confocal laser scanning microscope. mito-Keima fluorescence was imaged using dual excitation ratiometric pH measurements at 440 nm (pH 7.0) and 586 nm (pH 4.0) laser and 610 nm emission filters for neutral Keima (green) and acidic Keima (red). Mitophagy levels (%mitophagy) were defined as the number of pixels with a high red:green ratio divided by the total number of pixels. At least five images per group were examined to determine the mitophagy levels. For IB analysis, mitochondrial fractions were isolated using the Mitochondria Isolation Kit (TFS, 89874).

Mitochondrial analysis

Cells were stained with MitoTracker Red CMXRos dye (TFS, M46752) to specifically label mitochondria. Following staining, the cells were fixed with 4% PFA for 30 min on ice, and high-resolution mitochondrial images were acquired using Zeiss LSM980 Airyscan2 confocal laser scanning microscope. Images were captured at a resolution of 2048 × 2048 pixels with two-times averaging and quantitatively analyzed by NIH ImageJ, with the Mitochondria Analyzer plugin (http://sites.imagej.net/ACMito/) for semi-automated analysis of mitochondrial morphologies and

networks as previously defined and outlined [7]. The definitions of each parameter are as follows: 1) the form factor measures the shape of the mitochondria, with values closer to 1 indicating a more spherical form, 2) the mean area of mitochondria represents the average size of individual mitochondria within a cell.

Multiplex cytokine assay

The levels of SASP factors in media from WT and ΔUBR4 cells were quantified using a Luminex-based multiplex cytokine assay, conducted by LABISKOMA (Seoul, South Korea). Culture media were collected after 48 h, then centrifuged at 780 × g for 3 min to eliminate cellular debris. The resulting supernatants were stored at −80°C until further analysis.

GL13 staining with xenograft tissues

Sentragor™ (GL13) staining was performed following the manufacturer's instructions with slight modifications. Briefly, after deparaffinization and rehydration, and antigen retrieval, GL13 was applied dropwise onto the slides and incubated at RT for 1 h. Excess stain was then removed with 50% ethanol, followed by washes with 1 × PBS three times. The slides were subsequently incubated overnight at 4°C with an ABC-HRP kit. GL13 signals were detected with DAB with counterstaining with hematoxylin.

Statistical analysis

Statistical significance of differences between various groups was determined by two-tailed Student's t-test or one-way ANOVA followed by the Bonferroni post hoc test for most data (GraphPad Prism, ver. 9.3.1). Differences were considered statistically significant at $p < 0.05$. For TMT-MS, the Perseus software (ver. 1.6.15.0) was employed for statistical analysis, based on a threshold of a 1.5-fold change or higher, with a p-value less than 0.05. For TMA, statistical analysis was performed using SPSS (ver. 26.0) and GraphPad Prism. Associations between categorical data were assessed using Pearson's chi-square or Fisher's exact test. Overall survival was measured from the date of diagnosis to that of death from any cause, and progression-free survival was measured from the date of surgery to that of recurrence or metastasis. Survival analysis was performed using the Kaplan–Meier method with the log-rank test. Two-sided $p < 0.05$ was considered statistically significant for all analyses.

Supplementary References

1. Kim, H., et al., *Quantitative Proteomics Reveals Knockdown of CD44 Promotes Proliferation and Migration in Claudin-Low MDA-MB-231 and Hs 578T Breast Cancer Cell Lines.* J Proteome Res, 2021. **20**(7): p. 3720-3733.
2. Choi, W.H., et al., *Aggresomal sequestration and STUB1-mediated ubiquitylation during mammalian proteaphagy of inhibited proteasomes.* Proc Natl Acad Sci U S A, 2020. **117**(32): p. 19190-19200.
3. Perez-Riverol, Y., et al., *The PRIDE database and related tools and resources in 2019: improving support for quantification data.* Nucleic Acids Res, 2019. **47**(D1): p. D442-D450.
4. Love, M.I., W. Huber, and S. Anders, *Moderated estimation of fold change and dispersion for RNA-seq data with DESeq2.* Genome Biol, 2014. **15**(12): p. 550.
5. Choi, W.H., et al., *ECPAS/Ecm29-mediated 26S proteasome disassembly is an adaptive response to glucose starvation.* Cell Rep, 2023. **42**(7): p. 112701.
6. Lee, J.H., et al., *Facilitated tau degradation by USP14 aptamers via enhanced proteasome activity.* Sci Rep, 2015. **5**: p. 10757.
7. Valente, A.J., et al., *A simple ImageJ macro tool for analyzing mitochondrial network morphology in mammalian cell culture.* Acta Histochem, 2017. **119**(3): p. 315-326.
